# Supramolecular Interactions Modulate RNA:DNA Folding Observed via Nanopore Sensing

**DOI:** 10.1002/anie.202508917

**Published:** 2025-09-13

**Authors:** Thieme T. Schmidt, Max K. Earle, Gerardo Patiño‐Guillén, Yunxuan Li, Raluca‐Elena Alexii, Jeremy J. Baumberg, Ulrich F. Keyser, Casey M. Platnich

**Affiliations:** ^1^ Cavendish Laboratory University of Cambridge JJ Thomson Avenue Cambridge CB3 0US UK; ^2^ Present address: School of Chemistry Trinity College Dublin Dublin 2 Ireland

**Keywords:** Biophysics, Biosensing, RNA, Solid‐state nanopores, Supramolecular chemistry

## Abstract

DNA and RNA nanotechnology enables the precise assembly of molecular architectures, with applications in bioengineering, supramolecular chemistry, and sensing. The material properties of these biopolymers can be adjusted covalently or, more simply, through non‐covalent interactions with small molecules. Herein, we demonstrate the use of urea, an abundant and inexpensive small molecule, to modulate the stiffness of RNA:DNA hybrid nanostructures. Our results suggest that supramolecular interactions between urea and the A‐form‐like helix rigidify the hybrid polymers, as evidenced by both solid‐state nanopore measurements and atomic force microscopy. Nanopore sensing reveals that preparing topologically‐barcoded RNA:DNA hybrids in urea results in a 50% reduction in folded translocations. This decrease in folded events improves the proportion of useable data points, paving the way for the robust detection of low‐abundance RNA analytes at the single‐molecule level.

## Graphical abstract

Solid‐state nanopores enable the detection of RNA species at the single‐molecule level. To rapidly and accurately identify barcoded RNAs as they translocate the pore, it is imperative to reduce analyte folding. Here, we demonstrate that RNA's supramolecular interactions with urea inhibit folding, increasing the number of readable barcodes while decreasing the number of events required for RNA identification.

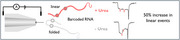

## Introduction

DNA and RNA nanotechnology provides a versatile toolbox for the programmable construction of nanoscale materials. Through the predictable Watson–Crick–Franklin base pairing interactions of nucleic acids, precise supramolecular topologies can be engineered using simple and robust self‐assembly principles.^[^ ^1^ ^]^ Extending beyond these base pairing interactions, small molecules may be leveraged to modulate the material properties of nucleic acids:^[^ ^2^, ^3^ ^]^ the exploration of these non‐covalent chemical approaches broadens the application space for DNA/RNA nanotechnology.

These recent advances in DNA/RNA nanotechnology, paired with emerging single‐molecule technologies,^[^ ^4^, ^5^, ^6^, ^7^ ^]^ open the door to new biosensing techniques for the quantification of low‐abundance biological analytes. For example, the detection of pathogenic RNA at early stages of infection can be a key step in the safe administration of therapeutics.^[^ ^8^ ^]^ Although current gold standard methods like quantitative polymerase chain reaction (qPCR) and RNA sequencing require enzymatic amplification steps, single‐molecule techniques, in conjunction with DNA/RNA self‐assembly, may be used to directly report on individual RNA strands, eliminating sequence biases and providing quantitative results.^[^ ^9^, ^10^ ^]^

As a single‐molecule technique, solid‐state nanopore sensing may be used to directly detect RNA and other biomolecules,^[^ ^11^, ^12^, ^13^, ^14^ ^]^ with recent publications demonstrating its use in identifying viral RNA,^[^ ^10^, ^15^, ^16^, ^17^ ^]^ probing RNA conformations,^[^ ^18^, ^19^, ^20^ ^]^ and detecting microRNAs,^[^ ^21^, ^22^, ^23^ ^]^ among other applications. In this sensing modality, a nanopore (here, a quartz glass nanopore with a diameter of 5–10 nm) is positioned between two reservoirs filled with an electrolyte solution, and a voltage is applied, inducing a current. When a charged analyte such as a nucleic acid is introduced to one of the reservoirs, this molecule will be electrophoretically driven through the nanopore according to its charge. During translocation of the analyte, ions are displaced from within the nanopore, causing a temporary change in the measured current. The characteristics of the resulting current signal provide information on the structure, conformation, and size of the analyte.^[^ ^12^, ^24^ ^]^

By combining the simple resistive pulse sensing principle with the programmability afforded by DNA/RNA nanotechnology, it is possible to create distinctive, three‐dimensional “barcodes,”^[^ ^25^, ^26^ ^]^ each with a recognizable current signature during nanopore translocation. Using previously established self‐assembly protocols, the barcode reshapes its target sequence into an “RNA ID” for direct nanopore identification even within complex mixtures of RNA sequences.^[^ ^27^ ^]^

In a solid‐state nanopore experiment, less than 10 unfolded events is sufficient to reach barcode consensus.^[^ ^28^ ^]^ One major hurdle, however, is analyte folding during translocation, which can obscure the barcode, rendering unambiguous readout difficult.^[^ ^29^ ^]^ To facilitate rapid readout with low numbers of reads (which is crucial for low‐abundance analytes and also reduces time to result), it is imperative to reduce folding such that target barcodes and their corresponding nucleic acid sequences can be readily identified.

**Figure 1:**
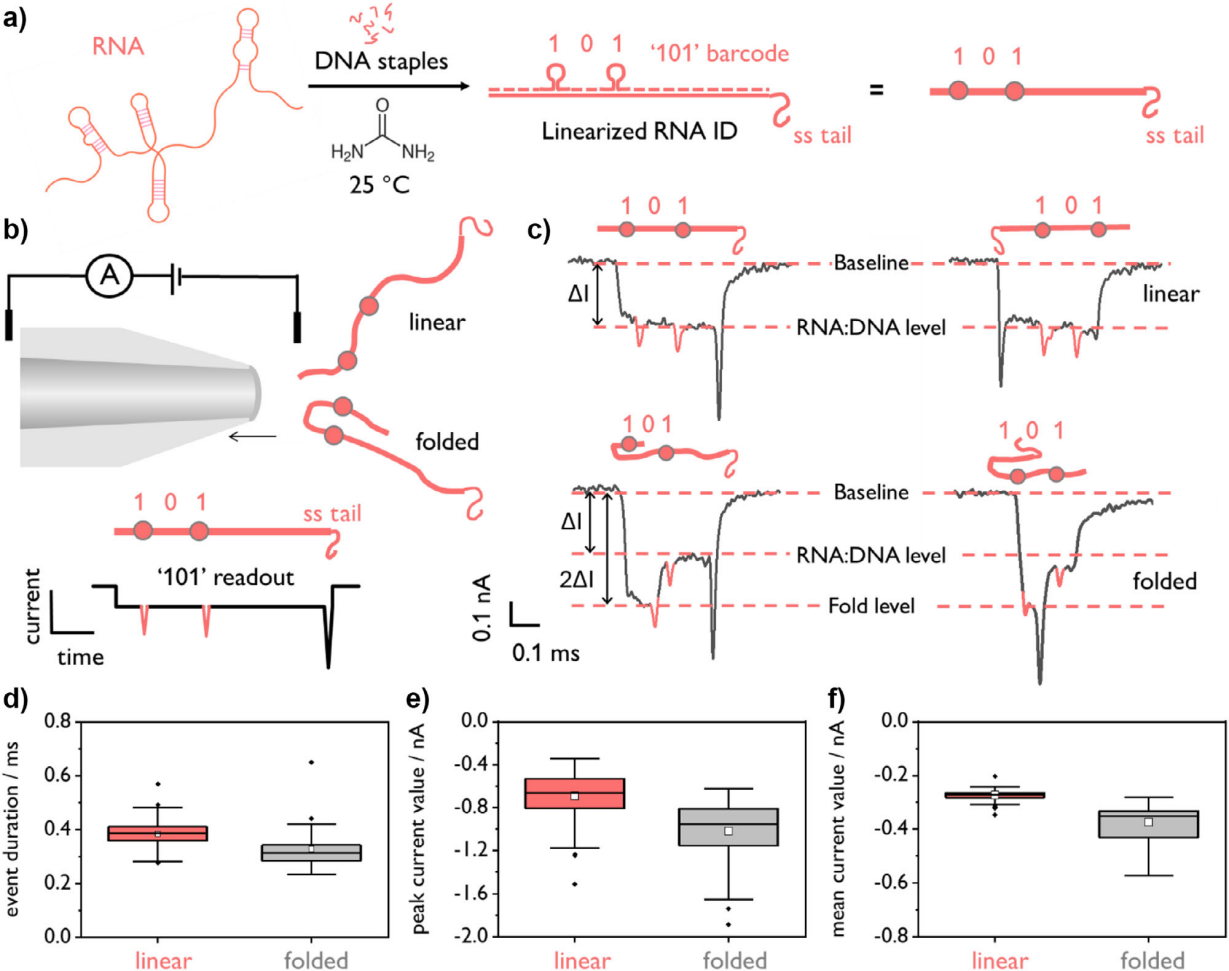
Overview of nanopore detection of folded versus linear RNA molecules. a) RNA's native secondary structure is disrupted via chemical annealing with complementary DNA staples at 25 °C (*t* = 12 h) in the presence of 5 M urea (100 mM LiCl, 10 mM Tris‐HCl, pH 7.5), resulting in the linearized RNA ID. b) Analytes may enter the nanopore in a linear (“single‐file”) manner, or folded. The hypothetical readout for the “101” RNA with single‐stranded tail is depicted. c) Real nanopore events showing the designed “101” barcode. Folds during translocation result in more complex current‐time trajectories, complicating readout. Note that the RNA is equally likely to enter from the 3′ or 5′ end, resulting in both types of events shown. d) Event durations for folded “101” RNA IDs are on average ∼ 17% shorter, reflecting their conformation. e) Peak current values are also ∼ 45% lower on average for folded species relative to linear ones. f) The mean current values for linear events are markedly more homogenous than for folded events, which are, on average, 33% greater in magnitude, as folds result in deeper current blockades. The data in (d) and (e) are from a single experiment and represent a total of 225 single‐molecule events.

Solid‐state nanopores allow various polymer arrangements to be captured, as the translocation time is markedly shorter than the relaxation time.^[^ ^30^ ^]^ Initially, the polymer diffuses randomly until the electric field of the nanopore pulls the molecule inside. The large size of the nanopore relative to the diameter of a double‐stranded nucleic acid allows for the detection of many conformations, including folds^[^ ^31^ ^]^ and knots.^[^ ^32^ ^]^ A simple, “single‐file” (or linear) translocation of double‐stranded DNA produces only a single current blockade level, I, while folded molecules will produce additional current levels with amplitudes of integer multiples of 2I (Figure 1). Folds are primarily observed at the start of the translocation event and can be distinguished based on their current levels.^[^ ^27^, ^28^ ^]^ Knots, on the other hand, give rise to sharp current blockages, characterized by their short duration and higher order (3I or more) current blockades. Knots are typically observed for very long (>10 kB), linearized, double‐stranded DNA molecules and are thus unlikely to be seen in the experiments presented here.^[^ ^26^ ^]^ By examining the number of current levels in each single‐molecule current‐time trace, it is thus possible to determine the tertiary structure of the polymer.^[^ ^33^ ^]^

Many strategies have been explored to reduce nucleic acid folding and knotting in solid‐state nanopores. For example, rigid DNA origami structures may be used to generate distinctive current signatures,^[^ ^34^ ^]^ though folding RNA into origami structures remains challenging. It is also possible to forcibly stretch target molecules prior to translocation^[^ ^15^, ^35^ ^]^—while effective, this strategy requires a more complex experimental setup. For glass nanopores, as employed here, folding may be mitigated by reducing the pore diameter to promote single‐file translocation.^[^ ^36^ ^]^ Unfortunately, smaller glass pores also suffer from reduced capture rates,^[^ ^36^ ^]^ extending the required measurement time. Furthermore, a reduction of the pore diameter increases the non‐specific interactions between pore and analyte, which can cause pore blockage.

Several other parameters may also be varied to modulate nucleic acid folding, namely: 1) cation concentration/identity, 2) applied voltage, and 3) pore charge (governed by pH).^[^ ^37^ ^]^ In the measurements presented here, we have maximized the negative charge of our glass nanopores by working at a pH of ∼9.4. High pH engenders a negatively charged surface, offering a repulsive force between the pore walls and the negatively charged phosphate groups of RNA/DNA. We also select Li^+^ as the cation, which has been shown to most effectively slow the translocation of nucleic acids in solid‐state nanopores.^[^ ^38^ ^]^ By slowing translocation, we increase the time between the bits in our barcode, thus promoting the error‐free decoding of the embedded information. Although the manipulation of other variables (cation concentration/voltage) may increase the proportion of unfolded events, there are significant drawbacks for each. For example, lowering the cation concentration from 4 M (as used here) to 1 M has been shown to reduce folding but also increases the speed of translocation, reducing the signal‐to‐noise ratio of the measurement.^[^ ^37^ ^]^ Lowering the voltage promotes the capture of linear species in glass nanopores, but also lowers the event rate, meaning that longer measurement times are required.^[^ ^39^ ^]^ As such, there is an ongoing need for simple, versatile methods to decrease folding without perturbing the nanopore measurement itself.

Here, we report a non‐covalent, chemical method to modulate the folding rate of RNA:DNA hybrids observed in glass nanopores without modifying any of the nanopore measurement parameters. We find that by preparing our DNA‐barcoded RNA structures in the presence of a high concentration of urea (5 M), folding is impeded even after buffer exchange. Although there is no added urea in the nanopore measurement itself, the duplexed RNA structures appear to sequester this small molecule, altering the folding behavior. Using atomic force microscopy (AFM), we find that the persistence length of the RNA:DNA hybrids increases when folded in urea, supporting our nanopore studies.

## Results and Discussion

We first used commercially available RNA from the MS2 bacteriophage (3569 nt) and designed a DNA barcode (“101”, Figure 1a) complementary to this sequence. Here, we employ a simple binary scheme based on the site‐specific hybridization of DNA dumbbells, but note that higher‐level encoding has been demonstrated with other DNA nanostructures.^[^ ^40^ ^]^ Using these established methods, it is theoretically possible to distinguish ∼ 5^10^ unique RNA IDs via nanopore sensing.^[^ ^41^ ^]^ Each “1” bit of the barcode consists of six consecutive DNA dumbbells, while the “0” bit is made of linear complement strands (Figure S1). A 300‐nt region at the RNA's 3′‐terminus is left unhybridized: the resulting deep current blockade^[^ ^42^ ^]^ allows us to determine the direction of translocation through the nanopore. For the “101” RNA ID, this tail does not promote translocation in any particular direction (Figure S2). To hybridize the complementary strands to the RNA, we employed our recently reported isothermal chemical annealing protocol.^[^ ^27^ ^]^ The RNA (20 nM) was incubated with its complementary DNA strands (100 nM) at *T* = 25 °C for 12 h in the presence of 100 mM LiCl and 5 M urea. The pH is held constant at 7.5 using tris‐HCl buffer (10 mM). We note here that the RNA IDs can be formed under these conditions in as little as 15 min—we have opted for a longer incubation period to enable complete hybridization of all DNA complements, as per our previous findings.^[^ ^27^ ^]^

After 12 h of incubation, the barcoded RNAs were separated from excess staples using Amicon filtration and exchanged into a urea‐free buffer (10 mM tris‐HCl, pH 8.0, 0.5 mM MgCl_2_). The concentrations of the resulting barcoded species were measured by Nanodrop and the formation of the barcodes was verified using agarose gel electrophoresis (Figure S3). The gel results also show that the RNA:DNA hybrid forms at the highest urea concentration tested—8 M.

Nanopore sensing was used to assess the translocation of folded and unfolded events for the RNA barcodes (Figure 1c–f). The characterization of the nanopores is shown in Figure S4. The sample was diluted to ∼ 300 pM in measurement buffer (10 mM tris‐EDTA (1 x TE), 4 M LiCl, pH 9.4, adjusted with LiOH) and introduced to the nanopore chip. The current through the nanopore was recorded while applying a voltage of 600 mV. From the resulting current‐time traces (Figure S5), single‐molecule events were extracted (Figure S6). The events were then examined, and the “101” barcodes were identified. These “Q. 101” events were then further divided into folded and linear categories based on the number of current levels observed (examples in Figure 1c and Figures S6 and S7).

Several event characteristics for each single‐molecule current‐time trajectory were recorded, including the total event duration, the peak event current (the minimum value for each event), and the mean event current. The differences in event duration (Figure 1d) are modest, and the peak current value is primarily dictated by the 300‐nt single‐stranded tail (Figure 1e). The mean current value differs significantly for the linear versus folded species because linear molecules have one dominant current level (the RNA:DNA level, as shown in Figure 1c). Folded molecules, on the other hand, exhibit several current levels with variable durations, giving rise to a broad distribution of mean currents. The mean current values thus provide a valuable metric for the assessment of folding between differing sample preparation methods (Figure 1f).

Next, a control sample was formed without the inclusion of urea (barcode “1111”). The RNA (20 nM) was heated with its complementary DNA strands (100 nM) to *T* = 70 °C for 1 min, then cooled to *T* = 4 °C over 45 min in the presence of 100 mM LiCl at pH 7.5. Control experiments were performed using thermal annealing, as incubating the RNA with DNA complements at 25 °C for 12 h without urea does not result in correctly formed barcodes, as previously demonstrated.^[^ ^27^ ^]^ Sample events for the 1111 barcode (formed via thermal annealing) are provided in the Supporting Information (Figure S8). To ensure no variations in pore shape or size, the urea‐annealed sample (barcode “101”) and control sample (barcode “1111”) were mixed in equimolar amounts in measurement buffer. This combined sample was then introduced to the same nanopore and measured simultaneously as described previously. The first 50 consecutive nanopore translocation events for this measurement are shown in Figure S9.

**Figure 2:**
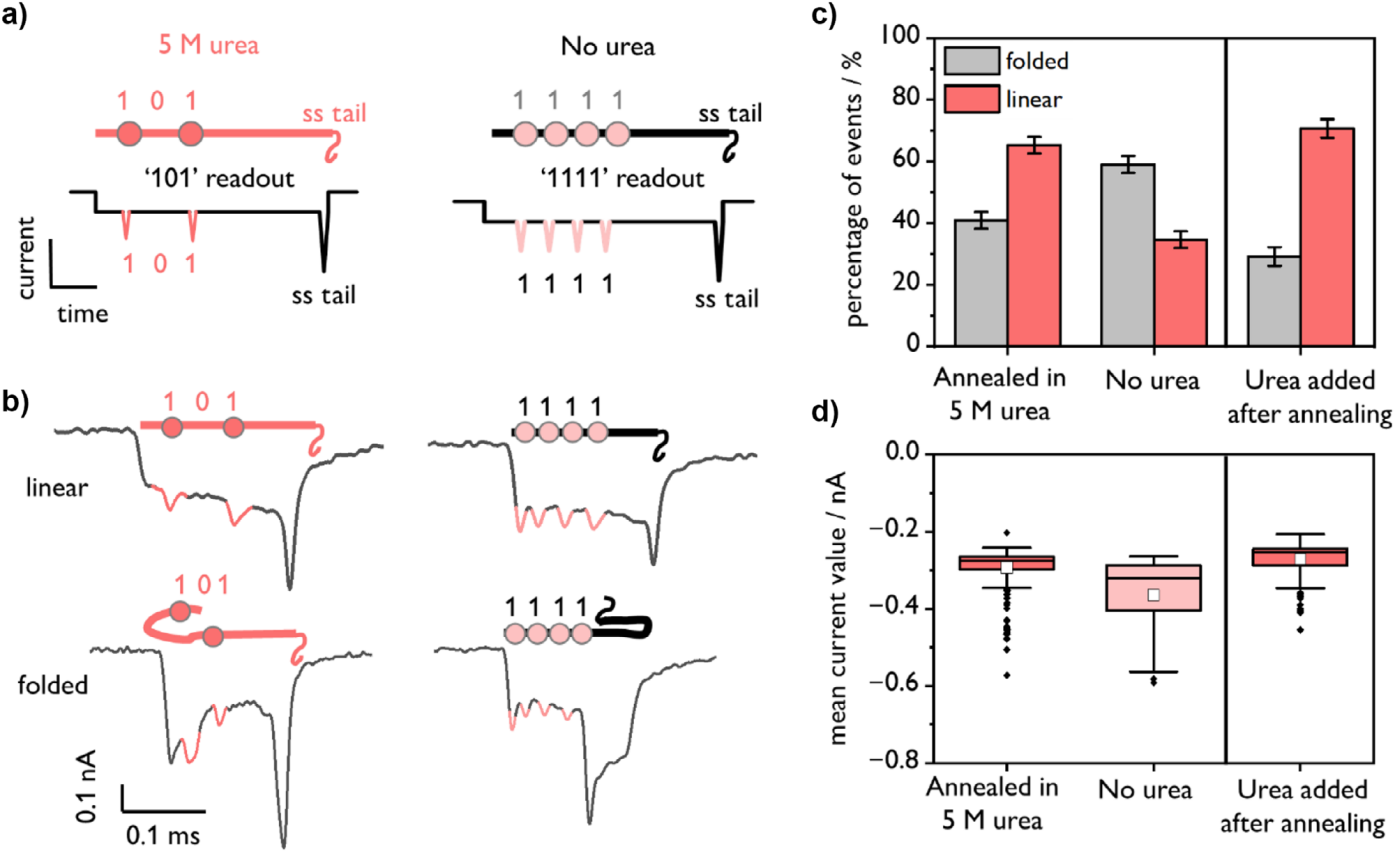
Comparison of nanopore events for RNA IDs annealed in the presence or absence of 5 M urea. “101” RNA IDs were formed isothermally in the presence of 5 M urea (*T* = 25 °C, *t* = 12 h), and “1111” RNA IDs formed via thermal annealing (70 to 4 °C over 45 min). Both samples were made in 100 mM LiCl, 10 mM Tris‐HCl, pH 7.5. a) Predicted nanopore current‐time trajectories for the “101” and “1111” RNA IDs based on the DNA barcode designs. b) Real nanopore events for the “101” and the “1111” RNA IDs. Example events are shown for both the linear and folded conformations. The two barcodes were prepared separately, then mixed in equimolar proportions and measured simultaneously in the same nanopore. c) When urea is employed in the folding procedure, the proportion of linear events increases from 34 (± 3)% to 65 (± 3)%. A total of 1000 single‐molecule events were measured for each of these experiments, which were performed in triplicate. The error bars represent the standard error. When urea is added to the sample after thermal annealing (rightmost panel), 71 (± 3)% of events (total events *N* = 729) bearing the “101” barcode are linear. d) The differences in the resulting translocations for the various annealing protocols are reflected in the mean current values for a single experiment, wherein the “101” barcodes (*N* = 228 identified barcodes), formed in urea, exhibit homogeneous mean current values, whereas a larger variation is observed when urea is not used (*N* = 58 identified barcodes). When urea is added after thermal annealing, (*N* = 205 identified barcodes), the distribution of mean current values closely resembles the sample annealed directly in urea. The colored boxes denote the 25%–75% range, the horizontal lines are the medians, the white squares are the means, and black points are outliers.

The predicted current versus time traces for the “101” and “1111” barcodes are depicted in Figure 2a, while sample events for both preparations are shown in Figure 2b. When 5 M urea is included in the annealing mixture (“101” barcode sample), the percentage of linear events nearly doubles relative to the thermally annealed sample, increasing from 34 (± 3)% to 65 (± 3)% (Figure 2c). The errors provided are the standard errors across three measurements in three different nanopores, to account for any differences in pore shape/size. When the urea concentration is halved (2.5 M), the proportion of linear translocation events remains above 60% (Figure S10).

Although events were sorted into folded/linear categories based on the number of current levels observed in each single‐molecule event, the differences in the translocation characteristics are also exemplified by the mean current values of each event as previously described (Figure 2d). The sample prepared in 5 M urea exhibits uniform mean current values, suggesting that the majority of events display a single main current level representing the linear RNA:DNA duplex. By contrast, the data is heterogeneous for the “1111” barcode formed via thermal annealing, reflecting the multiple current levels corresponding to folded events. Indeed, by comparing Figure 2d with Figure 1f, it is apparent that the mean current values for barcodes formed without urea closely resemble those of the folded “101” barcodes. Scatter plots of mean event current as a function of event duration can be found in Figure S11. It is also important to note that the difference we observe is not due to the barcodes themselves, as the same increase in unfolded events with urea persisted when the barcodes were swapped (Figure S12).

It is noteworthy that annealing the RNA barcode in the presence of urea results in significant differences in nanopore folding characteristics *even after buffer exchange*. We sought to ensure that the differences we observed were due to urea interacting directly with the RNA:DNA hybrid and not simply due to more efficient duplexing in urea. To this end, we conducted an experiment where urea was added to the RNA:DNA duplex after an initial thermal annealing procedure. The RNA (20 nM) was heated with its DNA complements (100 nM) to 70 °C for 1 min, then cooled to 4 °C over 45 min in the presence of 100 mM LiCl at pH 7.5. The sample was then Amicon filtered to remove excess staples (i.e., there is no possibility of “error correction”). Next, we subjected this filtered sample to a 2 h incubation in 5 M urea at *T* = 25 °C, then Amicon filtered a second time. Nanopore sensing was then conducted as previously described. Once again, a decrease in folded events was observed with a corresponding decreased magnitude of the mean current blockage (Figure 2c,d). This finding suggests that the addition of urea, whether before or after sample preparation, results in a greater proportion of unfolded events without perturbing any other parameters in the nanopore measurement. Interestingly, the results also imply that urea is bound to the RNA ID even after Amicon buffer exchange.

Stacking and NH–π interactions between urea and aromatic protein residues are well documented.^[^ ^43^ ^]^ These same supramolecular forces between the nucleobases and urea have been suggested using computational methods.^[^ ^43^, ^44^, ^45^, ^46^, ^47^ ^]^ Typically assumed to be responsible for denaturation, we wonder if these stacking and NH–π interactions can also, under specific conditions, exist within the RNA:DNA helix, to make the stack more rigid. Molecular dynamics have also revealed that, at 17 °C, 6 M urea primarily denatures alternative RNA configurations, favoring instead the formation of the A‐form helix.^[^ ^48^ ^]^ It is also worth noting here that the supramolecular interactions between small molecules and RNA are heavily salt dependent.^[^ ^49^ ^]^ For example, DNA origami structures have been found to persist at room temperature in urea concentrations as high as 6 M,^[^ ^50^ ^]^ with stability increasing with salt concentration.^[^ ^51^ ^]^ The molecular underpinnings require further study.

Although urea is typically thought of as a potent denaturant, there is still no consensus on the molecular origins of its effects.^[^ ^43^, ^44^, ^45^, ^46^, ^52^ ^]^ In fact, there are several reports of nucleic acid secondary structures that persist in, and are even stabilized by, high urea concentrations. For example, short (8 nt) single‐stranded DNA sequences containing only two GC pairs have been reported to produce hairpins that are stable even in 7 M urea.^[^ ^53^ ^]^ G‐quadruplexes (GQs) also exhibit unusual behavior in high concentrations of urea, with the melting temperature of single‐base loop GQs actually increasing with added urea.^[^ ^54^ ^]^ Stabilization of the GQ in molar concentrations of urea was found to be aided by small, monovalent cations,^[^ ^54^ ^]^ commensurate with the findings here.

The number of folded events is nearly halved when urea is employed in the barcode assembly, but the change in the duration of nanopore events is not significant (Figure S13), implying that the translocation velocity of the molecules is similar with and without urea. Our finding is in agreement with a previous report that showed that varying the persistence length of nucleic acids does not measurably alter their electrophoretic behavior within the nanopore.^[^ ^55^ ^]^ The capture rate and the noise are also unaffected by the urea, meaning that the nanopore measurement proceeds as normal, albeit with fewer folded events.

These features support the use of our methodology in RNA sensing assays. To demonstrate the feasibility of our chemical annealing protocol, we spiked MS2 RNA (10 nM) into a background of total human RNA (100 nM). Using 5 M urea and 50 nM of DNA staples, we formed the “101” barcode on MS2 using our previously described conditions. The barcode is readily identified in 20% of the total single‐molecule events (Figure S14). Although the MS2 was initially only at a ratio of 1:10 relative to the total RNA, it is likely enriched due to degradation/self‐cleavage of the single‐stranded RNA during room temperature incubation in 100 mM salt.^[^ ^56^, ^57^ ^]^

**Figure 3:**
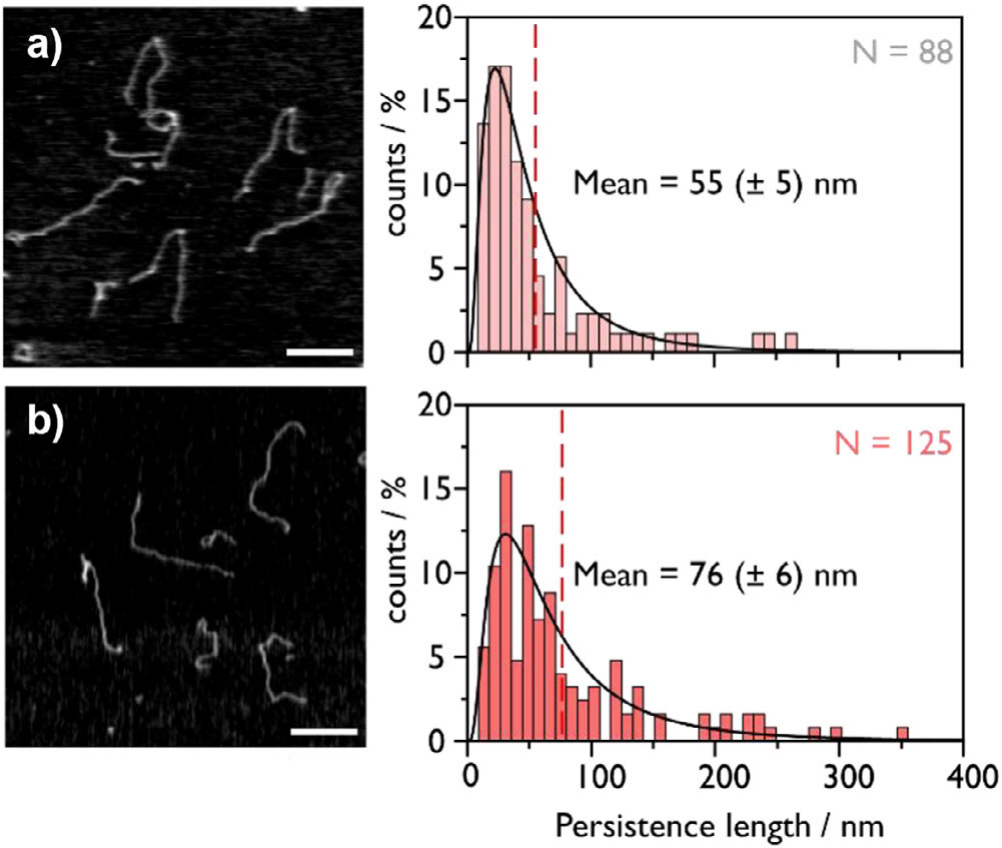
Atomic force microscopy of MS2 RNA duplexed with DNA staples in the absence a) and presence b) of 5 M urea. Scale bar = 400 nm. Persistence length histograms are fit with a log normal distribution—the tangent vector autocorrelation fitting reveals the difference in persistence length. Fitting parameters are detailed in Section S7.

To elucidate the origins of our observed changes in nanopore folding behavior, we performed AFM (Figure 3). AFM has previously been used to probe the persistence length of nucleic acids under various conditions, including in the presence of intercalating dyes,^[^ ^58^ ^]^ groove‐binding fluorophores,^[^ ^59^ ^]^ and DNA‐binding drugs.^[^ ^60^ ^]^ AFM has also been used to directly examine the morphological changes that lead to different folding behaviours in solid‐state nanopores.^[^ ^61^ ^]^

RNA:DNA hybrids were allowed to equilibrate on a mica surface in the presence of Mg^2+^ prior to rinsing and drying. This procedure ensures their relaxed conformation is measured.^[^ ^62^ ^]^ Details of AFM imaging and analysis are provided in Section S7 and Figure S15.^[^ ^63^, ^64^ ^]^ These experiments revealed an increase in the persistence length of ∼ 40%, shifting from 55 (± 5) to 76 (± 6) nm when duplexes were annealed using urea. Fitting was based on a 2D worm‐like chain model for equilibrated nucleic acid species (Section S7).^[^ ^62^, ^65^ ^]^ The rigidity of RNA:DNA hybrids is known to be dependent on salt concentration: a persistence length of 55 (± 5) nm for the thermally annealed (control) duplex is in agreement with literature values considering the 100 mM Li^+^ used in annealing.^[^ ^66^ ^]^

The change in morphology with urea annealing is evident in Figure 3 (with additional images in Figure S16). Mono‐intercalators, including ethidium bromide, chloroquine, and acridine, have previously been shown using AFM to increase the persistence length of DNA by 2–3 times.^[^ ^58^ ^]^ The DNA binding of some of these molecules, including ethidium bromide, has been probed with nanopores, but of much smaller diameters (∼3.5 nm), making folded conformations highly unlikely.^[^ ^67^ ^]^ Unlike ethidium bromide, urea does not appear to change the contour length of the RNA:DNA hybrid (Figure S17).^[^ ^68^ ^]^ Groove binders, including the dye SYBR Green I^[^ ^69^ ^]^ and the antibiotic distamycin A^[^ ^70^ ^]^ have been shown by optical tweezing experiments to increase the persistence length of nucleic acids without modifying the contour length.^[^ ^71^ ^]^ Although there is some experimental and computational evidence that urea may interact with the major and minor grooves of DNA,^[^ ^72^ ^]^ the binding mechanism for urea and the A‐form‐like RNA:DNA hybrid remains unclear.

A previous literature report interrogated the impact of ionic liquids on the conformations of DNA in solid‐state nanopores and found that certain bulky organic cations can reduce folding by increasing the DNA persistence length:^[^ ^61^ ^]^ our findings suggest that urea has a similar effect on RNA:DNA hybrids. As previously mentioned, this may be due to stacking/NH–π interactions between urea and the nucleobases of the A‐form‐like RNA:DNA helix.

Urea clearly modulates the persistence length of RNA:DNA hybrids based on our nanopore and AFM experiments. We next sought to examine whether double‐stranded DNA was equally affected or whether urea interacts preferentially with the RNA:DNA hybrid duplex. To test this, we designed 3‐bit barcodes for the M13 DNA sequence (7249 nt) commonly employed in DNA nanotechnology (Figure S18). As for the RNA experiments, we prepared two different barcodes for the 5 M urea assembly (“011”) and the control assembly (“001”), depicted in Figure S18. For the DNA barcodes, each “1” bit was formed from 11 consecutive dumbbells. A reference peak consisting of 12 dumbbells (and thus creating a slightly deeper current blockade) was also included to indicate directionality. After hybridization, Amicon filtration, and buffer exchange, these two species were mixed in a 1:1 stoichiometric ratio and introduced to a single nanopore.

Owing to the lower persistence length of DNA:DNA hybrids relative to RNA:DNA complements^[^ ^66^ ^]^ as well as the longer length of the DNA:DNA construct, a higher proportion of folded events were observed across all conditions as compared to the RNA:DNA hybrids. As shown in Figure S19, whether the DNA:DNA assembly was conducted chemically (with 5 M urea, 12 h at 25 °C) or thermally (0 M urea, annealed thermally from 70 to 4 °C over 45 min) the amount of folding remained constant (within the standard deviation between three measurements in separate nanopores). No difference in the mean current values is observed (Figure S19). Sample events are shown in Figures S20 and S21. The event durations and peak currents were also constant across the two different conditions (Figure S22). Furthermore, AFM did not reveal an increase in the persistence length in the presence of urea (Figures S23 and 24). These findings suggest that the supramolecular interactions of urea may be specific to the A‐form‐like helix in combination with the presence of RNA in the duplex. Future studies will focus on understanding the biophysical origins of these observations.

## Conclusion

In conclusion, our AFM and nanopore data show that urea impacts the persistence length of duplexed RNA in previously unforeseen ways. Contrary to the conventional wisdom that urea destabilizes nucleic acid duplexes, we observe that introducing urea into the annealing mixture actually produces duplexed RNA IDs in high yield. Moreover, the use of urea proffers advantages for nanopore sensing by decreasing the number of folded events. By increasing the proportion of recognizable current signatures, the number of total events required for reliable readout is lowered, enabling the quantification of low abundance species. Follow‐up studies will be focused on understanding the mechanism of urea complexation by RNA and its impacts on the mechanical properties of nucleic acid duplexes, including the use of single‐molecule force experiments. The effects of other small molecules, including intercalating dyes and aromatic drugs, may also be investigated to probe the origins of the increased persistence lengths we observe. By expanding the use of non‐covalent interactions, the translocation behaviors of polymers through nanopores may be modulated, resulting in simpler decoding with lower numbers of reads required to reach a consensus. Although here we focused on glass nanopores, it is possible that these methods could be extended to the larger protein nanopores that have recently been reported.^[^ ^73^ ^]^ Beyond nanopore sensing, our study expands on the use of inexpensive, small molecules to manipulate the material properties of biopolymers, with applications in nanomaterials, supramolecular assembly, and fundamental biochemistry.

## Conflict of Interests

The authors declare no conflict of interest.

## Supporting information



Supporting Information

## Data Availability

The data that support the findings of this study are available from the corresponding author upon reasonable request.

## References

[1] N. C. Seeman , H. F. Sleiman , Nat. Rev. Mater. 2017, 3, 17068.

[2] Q. Li , J. Zhao , L. Liu , S. Jonchhe , F. J. Rizzuto , S. Mandal , H. He , S. Wei , H. F. Sleiman , H. Mao , C. Mao , Nat. Mater. 2020, 19, 1012–1018. 32661383 10.1038/s41563-020-0728-2 PMC7732259

[3] C. Lachance‐Brais , M. Rammal , J. Asohan , A. Katolik , X. Luo , D. Saliba , A. Jonderian , M. J. Damha , M. J. Harrington , H. F. Sleiman , Adv. Sci. 2023, 10, e2205713. 10.1002/advs.202205713 PMC10131789 36752390

[4] R. Tsukanov , T. E. Tomov , M. Liber , Y. Berger , E. Nir , Acc. Chem. Res. 2014, 47, 1789–1798. 24828396 10.1021/ar500027d

[5] C. M. Platnich , F. J. Rizzuto , G. Cosa , H. F. Sleiman , Chem. Soc. Rev. 2020, 49, 4220–4233. 32538403 10.1039/c9cs00776h

[6] A. Rajendran , M. Endo , H. Sugiyama , Angew. Chem. Int. Ed. 2012, 51, 874–890. 10.1002/anie.201102113 22121063

[7] F. Li , J. Li , B. Dong , F. Wang , C. Fan , X. Zuo , Chem. Soc. Rev. 2021, 50, 5650–5667. 33729228 10.1039/d0cs01281e

[8] S. A. Byron , K. R. Van Keuren‐Jensen , D. M. Engelthaler , J. D. Carpten , D. W. Craig , Nat. Rev. Genet. 2016, 17, 257–271. 26996076 10.1038/nrg.2016.10 PMC7097555

[9] L. Grabenhorst , M. Pfeiffer , T. Schinkel , M. Kümmerlin , G. A. Brüggenthies , J. B. Maglic , F. Selbach , A. T. Murr , P. Tinnefeld , V. Glembockyte , Nat. Nanotechnol. 2025, 20, 303–310. 39511326 10.1038/s41565-024-01804-0

[10] F. Bošković , J. Zhu , R. Tivony , A. Ohmann , K. Chen , M. F. Alawami , M. Đorđević , N. Ermann , J. Pereira‐Dias , M. Fairhead , M. Howarth , S. Baker , U. F. Keyser Nat. Nanotechnol. 2023, 18, 290–298. 36646828 10.1038/s41565-022-01287-x PMC10020084

[11] K.‐L. Chen , R.‐J. Yu , C.‐B. Zhong , Z. Wang , B.‐K. Xie , H. Ma , M. Ao , P. Zheng , A. G. Ewing , Y.‐T. Long , Angew. Chem. Int. Ed. 2024, 63, e202406677. 10.1002/anie.202406677 38825572

[12] R.‐J. Yu , Y.‐L. Ying , R. Gao , Y.‐T. Long , Angew. Chem. Int. Ed. 2019, 58 (12), 3706–3714. 10.1002/anie.201803229 30066493

[13] K. Chuah , Y. Wu , S. R. C. Vivekchand , K. Gaus , P. J. Reece , A. P. Micolich , J. J. Gooding , Nat. Commun. 2019, 10, 2109. 31068594 10.1038/s41467-019-10147-7 PMC6506515

[14] Y. M. N. D. Y. Bandara , K. J. Freedman , J. Am. Chem. Soc. 2024, 146, 3171–3185. 38253325 10.1021/jacs.3c11044

[15] E. Atas , A. Singer , A. Meller , Electrophoresis 2012, 33, 3437–3447. 23109189 10.1002/elps.201200266 PMC3773941

[16] K. Sethi , G. P. Dailey , O. K. Zahid , E. W. Taylor , J. A. Ruzicka , A. R. Hall , ACS Nano 2021, 15, 8474–8483. 33914524 10.1021/acsnano.0c10887 PMC8801185

[17] H. Wang , R. Zhao , B. Zhang , Y. Xiao , C. Yu , Y. Wang , C. Yu , Y. Tang , Y. Li , B. Lu , B. Li , Angew. Chem. Int. Ed. 2025, 64, e202423473. 10.1002/anie.202423473 39804233

[18] C. Chau , F. Marcuccio , D. Soulias , M. A. Edwards , A. Tuplin , S. E. Radford , E. Hewitt , P. Actis , ACS Nano 2022, 16, 20075–20085. 36279181 10.1021/acsnano.2c08312 PMC9798860

[19] S. Namani , K. Kavetsky , C.‐Y. Lin , S. Maharjan , H. B. Gamper , N.‐S. Li , J. A. Piccirilli , Y.‐M. Hou , M. Drndic , ACS Nano 2024, 18, 17240–17250. 38906834 10.1021/acsnano.4c04625 PMC12032626

[20] C. Shasha , R. Y. Henley , D. H. Stoloff , K. D. Rynearson , T. Hermann , M. Wanunu , ACS Nano 2014, 8, 6425–6430. 24861167 10.1021/nn501969r PMC4729693

[21] M. Wanunu , T. Dadosh , V. Ray , J. Jin , L. McReynolds , M. Drndić , Nat. Nanotechnol. 2010, 5, 807–814. 20972437 10.1038/nnano.2010.202

[22] E. Beamish , V. Tabard‐Cossa , M. Godin , Nanoscale 2020, 12, 17833–17840. 32832949 10.1039/d0nr03878d

[23] O. K. Zahid , F. Wang , J. A. Ruzicka , E. W. Taylor , A. R. Hall , Nano Lett. 2016, 16, 2033–2039. 26824296 10.1021/acs.nanolett.6b00001 PMC5367926

[24] L. Xue , H. Yamazaki , R. Ren , M. Wanunu , A. P. Ivanov , J. B. Edel , Nat. Rev. Mater. 2020, 5, 931–951.

[25] Y. Li , S. C. Meng , Y. Wang , C. M. Platnich , M. K. Earle , E. Mylona , P. Naydenova , S. Baker , J. Zhu , U. F. Keyser , Nat. Nanotechnol. 2025, 10.1038/s41565-025-01965-6. PMC12534182 40579472

[26] G. Patiño‐Guillén , J. Pešović , M. Panić , D. Savić‐Pavićević , F. Bošković , U. F. Keyser , Nat. Commun. 2024, 15, 1699. 38402271 10.1038/s41467-024-45968-8 PMC10894232

[27] C. M. Platnich , M. K. Earle , U. F. Keyser , J. Am. Chem. Soc. 2024, 146, 12919–12924. 38691627 10.1021/jacs.4c03753 PMC11099964

[28] N. A. W. Bell , U. F. Keyser , Nat. Nanotechnol. 2016, 11, 645–651. 27043197 10.1038/nnano.2016.50

[29] Z. Roelen , K. Briggs , V. Tabard‐Cossa , ACS Sens. 2023, 8, 2809–2823. 37436112 10.1021/acssensors.3c00751 PMC10913896

[30] K. Chen , I. Jou , N. Ermann , M. Muthukumar , U. F. Keyser , N. A. W. Bell , Nat. Phys. 2021, 17, 1043–1049.

[31] A. J. Storm , J. H. Chen , H. W. Zandbergen , C. Dekker , Phys. Rev. E 2005, 71, 051903. 10.1103/PhysRevE.71.051903 16089567

[32] C. Plesa , D. Verschueren , S. Pud , J. Van Der Torre , J. W. Ruitenberg , M. J. Witteveen , M. P. Jonsson , A. Y. Grosberg , Y. Rabin , C. Dekker , Nat. Nanotechnol. 2016, 11, 1093–1097. 27525473 10.1038/nnano.2016.153

[33] J. Li , M. Gershow , D. Stein , E. Brandin , J. A. Golovchenko , Nat. Mater. 2003, 2, 611–615. 12942073 10.1038/nmat965

[34] M. Raveendran , A. J. Lee , R. Sharma , C. Wälti , P. Actis , Nat. Commun. 2020, 11, 4384. 32873796 10.1038/s41467-020-18132-1 PMC7463249

[35] K. Briggs , G. Madejski , M. Magill , K. Kastritis , H. W. de Haan , J. L. McGrath , V. Tabard‐Cossa , Nano Lett. 2018, 18, 660–668. 29087723 10.1021/acs.nanolett.7b03987 PMC5814347

[36] M. Wanunu , J. Sutin , B. McNally , A. Chow , A. Meller , Biophys. J. 2008, 95, 4716–4725. 18708467 10.1529/biophysj.108.140475 PMC2576395

[37] N. Ermann , N. Hanikel , V. Wang , K. Chen , N. E. Weckman , U. F. Keyser , J. Chem. Phys. 2018, 149, 163311. 30384733 10.1063/1.5031010

[38] S. W. Kowalczyk , D. B. Wells , A. Aksimentiev , C. Dekker , Nano Lett. 2012, 12, 1038–1044. 22229707 10.1021/nl204273h PMC3349906

[39] A. Y. Grosberg , Y. Rabin , J. Chem. Phys. 2010, 133, 165102. 21033823 10.1063/1.3495481

[40] J. Zhu , N. Ermann , K. Chen , U. F. Keyser , Small 2021, 17, 2100711. 10.1002/smll.202100711 34133074

[41] F. Bošković , U. F. Keyser , Nat. Chem. 2022, 14, 1258–1264. 36123450 10.1038/s41557-022-01037-5

[42] M. van den Hout , G. M. Skinner , S. Klijnhout , V. Krudde , N. H. Dekker , Small 2011, 7, 2217–2224. 21638785 10.1002/smll.201100265

[43] S. Goyal , A. Chattopadhyay , K. Kasavajhala , U. D. Priyakumar , J. Am. Chem. Soc. 2017, 139, 14931–14946. 28975780 10.1021/jacs.7b05463

[44] B. J. Bennion , V. Daggett , Proc. Natl. Acad. Sci. USA 2003, 100, 5142–5147. 12702764 10.1073/pnas.0930122100 PMC154312

[45] K. Kasavajhala , S. Bikkina , I. Patil , A. D. MacKerell , U. D. Priyakumar Jr. , J. Phys. Chem. B 2015, 119, 3755–3761. 25668757 10.1021/jp512414f PMC4352126

[46] S. Raghunathan , T. Jaganade , U. D. Priyakumar , Biophys. Rev. 2020, 12, 65–84. 32067192 10.1007/s12551-020-00620-9 PMC7040157

[47] G. Suresh , S. Padhi , I. Patil , U. D. Priyakumar , Biochemistry 2016, 55, 5653–5664. 27657980 10.1021/acs.biochem.6b00309

[48] J. C. Miner , A. E. García , J. Phys. Chem. B 2017, 121, 3734–3746. 28181434 10.1021/acs.jpcb.6b10767

[49] C. S. Chow , F. M. Bogdan , Chem. Rev. 1997, 97, 1489–1514. 11851457 10.1021/cr960415w

[50] S. Ramakrishnan , G. Krainer , G. Grundmeier , M. Schlierf , A. Keller , Nanoscale 2016, 8, 10398–10405. 27142120 10.1039/c6nr00835f

[51] S. Ramakrishnan , G. Krainer , G. Grundmeier , M. Schlierf , A. Keller , Small 2017, 13, 1702100. 10.1002/smll.201702100 29024433

[52] I. P. de Oliveira , L. Martínez , Phys. Chem. Chem. Phys. 2020, 22, 354–367. 10.1039/c9cp05196a 31815262

[53] I. Hirao , Y. Nishimura , T. Naraoka , K. Watanabe , Y. Arata , K.‐i. Miura , Nucleic Acids Res. 1989, 17, 2223–2231. 2704619 10.1093/nar/17.6.2223 PMC317592

[54] N. Tariq , C. Xu , J. Wang , T. Kume , R. B. Macgregor , Biophys. Chem. 2023, 299, 107043. 37285661 10.1016/j.bpc.2023.107043

[55] F. Bošković , C. Maffeo , G. Patiño‐Guillén , R. Tivony , A. Aksimentiev , U. F. Keyser , ACS Nano 2024, 18, 15013–15024. 38822455 10.1021/acsnano.4c01466 PMC11171748

[56] W. Yang , Q. Rev. Biophys. 2011, 44, 1–93. 20854710 10.1017/S0033583510000181 PMC6320257

[57] A. T. Perrotta , M. D. Been , Biochemistry 2006, 45, 11357–11365. 16981696 10.1021/bi061215+

[58] J. Tibbs , S. M. A. Tabei , T. E. Kidd , J. P. Peters , J. Phys. Chem. B 2020, 124, 8572–8582. 32941733 10.1021/acs.jpcb.0c06867

[59] A. Japaridze , A. Benke , S. Renevey , C. Benadiba , G. Dietler , Macromolecules 2015, 48, 1860–1865.

[60] V. Cassina , D. Seruggia , G. L. Beretta , D. Salerno , D. Brogioli , S. Manzini , F. Zunino , F. Mantegazza , Eur. Biophys. J. 2011, 40, 59–68. 20882274 10.1007/s00249-010-0627-6

[61] K.‐B. Jeong , K. Luo , M.‐C. Lim , J.‐Y. Jung , J.‐S. Yu , K.‐B. Kim , Y.‐R. Kim , Small 2018, 14, 1801375.

[62] C. Rivetti , M. Guthold , C. Bustamante , J. Mol. Biol. 1996, 264, 919–932. 9000621 10.1006/jmbi.1996.0687

[63] M. A. Beuwer , M. F. Knopper , L. Albertazzi , D. van der Zwaag , W. G. Ellenbroek , E. W. Meijer , M. W. J. Prins , P. Zijlstra , Polym. Chem. 2016, 7, 7260–7268.

[64] T. L. Hawkins , M. Mirigian , J. Li , M. S. Yasar , D. L. Sackett , D. Sept , J. L. Ross , Cell Mol. Bioeng. 2012, 5, 227–238.

[65] F. J. Rizzuto , C. M. Platnich , X. Luo , Y. Shen , M. D. Dore , C. Lachance‐Brais , A. Guarné , G. Cosa , H. F. Sleiman , Nat. Chem. 2021, 13, 843–849. 34373598 10.1038/s41557-021-00751-w

[66] C. Zhang , H. Fu , Y. Yang , E. Zhou , Z. Tan , H. You , X. Zhang , Biophys. J. 2019, 116, 196–204. 30635125 10.1016/j.bpj.2018.12.005 PMC6350079

[67] M. Wanunu , J. Sutin , A. Meller , Nano Lett. 2009, 9, 3498–3502. 19585985 10.1021/nl901691v PMC2871189

[68] I. D. Vladescu , M. J. McCauley , I. Rouzina , M. C. Williams , Phys. Rev. Lett. 2005, 95, 158102. 16241765 10.1103/PhysRevLett.95.158102

[69] S. Husale , W. Grange , M. Hegner , Single Mole. 2002, 3, 91–96. .

[70] I. Tessmer , C. G. Baumann , G. M. Skinner , J. E. Molloy , J. G. Hoggett , S. J. B. Tendler , S. Allen , J. Mod. Opt. 2003, 50, 1627–1636.

[71] A. Sischka , K. Toensing , R. Eckel , S. D. Wilking , N. Sewald , R. Ros , D. Anselmetti , Biophys. J. 2005, 88, 404–411. 15516529 10.1529/biophysj.103.036293 PMC1305017

[72] S. Sarkar , P. C. Singh , Biochimica et Biophysica Acta (BBA) – General Sub. 2020, 1864, 129498. 10.1016/j.bbagen.2019.129498 31785326

[73] W. Chanakul , A. Mukhopadhyay , S. Awasthi , A. D. Protopopova , A. Ianiro , M. Mayer , ACS Nano 2025, 19, 5240–5252. 39871506 10.1021/acsnano.4c11666 PMC11823641

