# Supplementary material for: Supramolecular Interactions Modulate RNA:DNA Folding Observed via Nanopore Sensing
Source: Angew Chem Int Ed Engl. 2025 Sep 13;64(45):e202508917. doi: 10.1002/anie.202508917 (PMC12582014; doi:10.1002/anie.202508917)
Supplement: Supplementary file 1 — Supporting Information [file ANIE-64-e202508917-s001.pdf]

Supporting Information

Supramolecular Interactions Modulate RNA:DNA Folding Observed via Nanopore Sensing

Thieme T. Schmidt, Max K. Earle, Gerardo Patiño-Guillén, Yunxuan Li, Raluca-Elena Alexii, Jeremy J. Baumberg, Ulrich F. Keyser*, Casey M. Platnich*

Correspondence to: ufk20@cam.ac.uk, cp769@cam.ac.uk

Cavendish Laboratory, University of Cambridge, CB3 0HE Cambridge, United Kingdom

Contents:

1. Materials
2. Assembly of hybrids
3. Native agarose gel electrophoresis
4. Nanopore fabrication
5. Nanopore measurements
6. Estimation of nanopore size
7. Nanopore data analysis
8. Atomic force microscopy
9. Supplementary figures
10. Oligonucleotide sequences
11. References

1. Materials.

Glass quartz capillaries with filament (inner diameter 0.2 mm, outer diameter 0.5 mm) were purchased from Sutter Instruments (California, USA). Mica discs (10 mm, V1) for atomic force microscopy (AFM) were purchased from Agar Labs. AFM tips were purchased from Bruker (OTESPA-R3).

DNA oligonucleotides were purchased from Integrated DNA Technologies as OligoPools (unmodified, 50 pmol scale). The oligo sequences are listed in Tables S1-S4. MS2 RNA (3569 nt in length) was purchased from Roche and used without further purification. Single-stranded circular m13mp18 7249 nt in length was purchased from Guild Biosciences (foundation m13). PDMS used in the fabrication of nanopore chips is Sylgard 184 silicone elastomer kit (The Dow Chemical Company, MI, USA). All water is DEPC-treated nuclease-free water (Merck). Stock 20x tris buffer (nuclease-free) was purchased from Fisher Scientific. All buffers were filtered with 0.22 µm Millipore syringe filter units (Merck).

2. Assembly of hybrids.

Assembly was performed at 20 nM of the RNA strand with DNA complements added at a 5x excess (100 nM). 1 x Tris-HCl (pH 7.5) was used to buffer and LiCl (100 mM final) was used to screen the negative charges of the phosphate backbone. Urea was added to a final concentration of 5 M unless otherwise indicated. Samples annealed without the inclusion of urea were heated to 70 °C and cooled over the course of 45 min, to denature any secondary structure. The sample was then mixed by flicking, microcentrifuged, and placed in a thermocycler (Applied Biosystems™ ProFlex™ PCR System, 3 x 32-well) to incubate at a given temperature for a controlled time interval, after which the temperature was held at 4 °C until ready to filter. The sample was filtered (100 kDa Amicon filter, Merck) and its concentration was measured in a Thermo Scientific Nanodrop™ 2000 Spectrophotometer.

3. Native agarose gel electrophoresis.

Agarose gel electrophoresis (0.8% w/v agarose) was conducted using a BioRad Sub-Cell GT electrophoresis cell with 1 x TBE (with 0.05% sodium hypochlorite solution) as the running buffer. Gels were run for 2.5 hours at 70 V on ice. Samples are prepared to 150 ng RNA in 10 uL for loading and consist of the sample, 1 x TBE buffer and 1 x purple loading dye (no SDS). The ladder used is indicated for each gel. After running, gels were stained using GelRed (Biotium) and imaged using the GelDoc-It™ (UVP). Gel images were processed using Fiji (ImageJ) by inverting the grayscale and subtracting the homogenous background with 100-150 pixels rolling ball.

4. Nanopore fabrication.

Quartz glass capillaries were pulled to the desired diameter (~10 nm) using a laser-heated pipette puller (P-2000, Sutter Instrument, California, USA). The parameters used were: HEAT=475, FIL=0, VEL=25, DEL=170, PUL=225. Please refer to the P-2000 manual for the

explanation of the parameters. After pulling, nanopore were cut to length and positioned within a custom polydimethylsiloxane (PDMS) chip with 8 pores per chip, which was then plasma bonded (Femto, Diener Electronic, Germany) to a glass slide. PDMS was used to seal with pores within the chip and baked at 120 °C for 2 hours for curing. After baking, chips are placed in the plasma cleaner for 5 minutes to ensure a hydrophilic surface layer. 1 x TE buffer with 4 M LiCl with a pH of 9.4 (adjusted with LiOH) was then added to the central reservoir as well as the outer chambers.

Samples for nanopore measurements were diluted to ~ 300 pM in 1 x TE, 4 M Li (pH 9.4) and injected into the central reservoir.

5. Nanopore measurements.

An Axopatch 200B (Molecular Devices, CA, USA) was used to perform nanopore measurements. The signal was filtered with an external Bessel filter (Frequency Devices) at 50 kHz and digitized at a 250 kHz sampling rate with a data card (PCI-6251, National Instruments). Two Ag/AgCl electrodes were prepared by curing 1-mm Ag wires in a 10% solution of NaClO. These were then inserted into the central reservoir (cis) and the outer chamber (trans) to create an electrical circuit across the nanopore. Current-voltage curves were measured from -600 mV to 600 mV to estimate nanopore size prior to measurements. Approximate diameters were calculated from their conductance as previously described. Pores with a maximum current of ~ 10 nA and a root-mean-square (RMS) noise below 7 pA were selected for measurements. The current-voltage curves and noise levels for nanopores used in this work are given in Figure S4.

6. Nanopore size estimation.

Nanopore diameters were estimated using methods previously described by our group.[1, 2] The resistance of the nanopore has two components: the resistance of the pore itself R_{pore} and the access resistance, R_{acc}.

$$R = R_{pore} + R_{acc}$$

Considering the resistivity of the electrolytic solution ρ, the pore length *L* and the diameters of the cis and trans pore apertures, D_{cis} and D_{trans}, this can be rewritten as:

$$R = \rho \frac{4L}{\pi D_{trans} D_{cis}} + \rho \left(\frac{1}{2D_{trans}} + \frac{1}{2D_{cis}} \right)$$

The diameter of the pore D_{cis} was calculated using the value of the ionic current under a 600 mV potential. We assume D_{trans} = 200 µm and L= 950 µm for our glass nanopores based on previous electron microscopy images.[1] We assume a conductivity of 15.5 Sm^{-1} for 4 M LiCl (in which all experiments were conducted for this manuscript.

7. Nanopore data analysis.

The data was analysed using an in-house peak finding algorithm in LabVIEW. Individual events were isolated by thresholding by duration and current drop. To ensure that all events are captured from the raw data, a low minimum event duration of 0.05 ms was used as the lower threshold. A lower bound for current peak depth was set at -0.1 nA. No upper bounds were set on event duration or current peak depth, such that any higher order structures would also be visualised. From the isolated events, we can then discriminate between folded and unfolded events, as well as determining the barcode by identifying the downward peaks within each event.

8. Atomic force microscopy.

AFM imaging was performed using an MFP-3DAFM System from Asylum/Oxford Instruments. The imaging was conducted in air using non-contact mode. Samples were diluted to a concentration of ~5 nM in 0.5 mM $MgCl_2$. 10 µL of this solution was pipetted directly onto freshly cleaved mica and incubated for 60 s. The mica surface was then rinsed with 3 x 50 µL of nuclease-free water then blown dry with a stream of nitrogen. Image visualization and analysis were carried out using Gwyddion and ImageJ/Fiji software.

Each DNA or RNA duplex was manually identified and traced using the NeuronJ plugin from ImageJ/Fiji. To allow for subsequent data analysis of smaller contour segments, the traced contours are smoothed using cubic spline interpolation. This smoothing ensures more reliable tangent vector calculations over short distances, minimizing artifacts from imaging noise or manual tracing inaccuracies.

The persistence length (L_p) of each trace was calculated by analysing the decay rate of the tangent-tangent correlation function.[3, 4] The tangent-tangent correlation was computed as the cosine correlation function:

$$\langle t(s) \cdot t(s') \rangle = \langle \cos\theta\,(s, s') \rangle = e^{-\frac{|s-s'|}{L_p}}$$

Where $t(s)$ and $t(s')$ are the tangent vectors at contour positions s and s', respectively and $\theta(s, s')$ is the angle between these two positions. The exponential decay of the correlation as a function of the contour separation $|s - s'|$ allows for the extraction of L_p through curve fitting. See Figure S15 for a graphical explanation.

To obtain a statistical representation of the persistence lengths, a histogram of the apparent persistence lengths was constructed. The mean and standard deviation of the persistence lengths are determined by fitting a log-normal distribution to this histogram.

This model assumes that nucleic acid molecules deposited onto freshly cleaved mica in the presence of low concentrations of divalent salts are able to equilibrate and thus behave as ideal worm-like chains in two dimensions.

9. Supplementary figures.

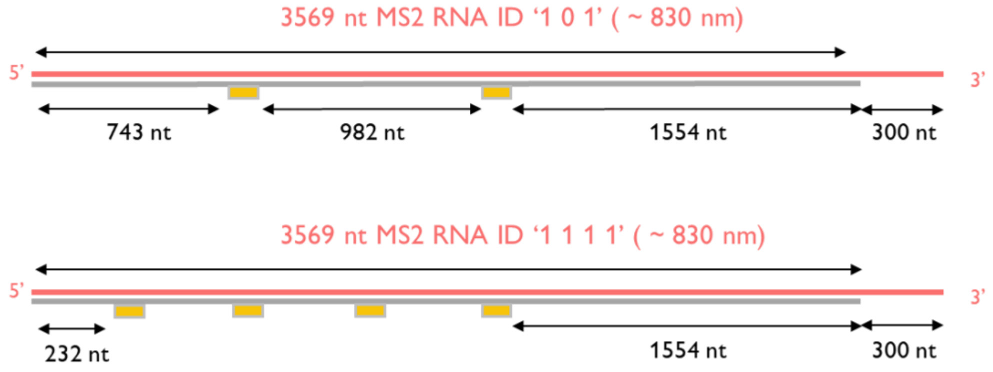

Figure S1. Designs for RNA IDs. Approximate lengths for the constructs are given assuming an A-form helical geometry.

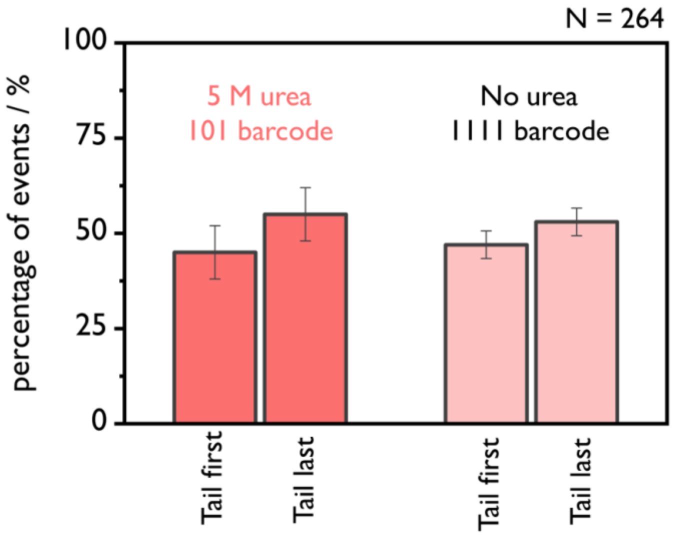

Figure S2. The number of events translocating with the single-stranded tail entering the pore and entering the pore last are roughly equal. MS2 RNA was used to form two separate barcodes which were annealed separately then mixed in equimolar amounts prior to introduction to the same nanopore. The data represents a total of 264 single-molecule events from one nanopore measurement. "1 0 1" RNA IDs were formed isothermally (T = 25 °C, t = 12 h) in the presence of 5 M urea while "1 1 1 1" RNA IDs were formed via thermal annealing (ramp from 70 °C to 4 °C over 45 minutes). Both hybridization mixtures contained 100 mM LiCl, 10 mM Tris-HCl, pH 7.5. Error bars represent the standard deviation based on a binomial distribution. Using the binomial test we find the p values of 0.78 and 0.13 for the 101 and 1111 barcodes respectively, making them not significantly different.

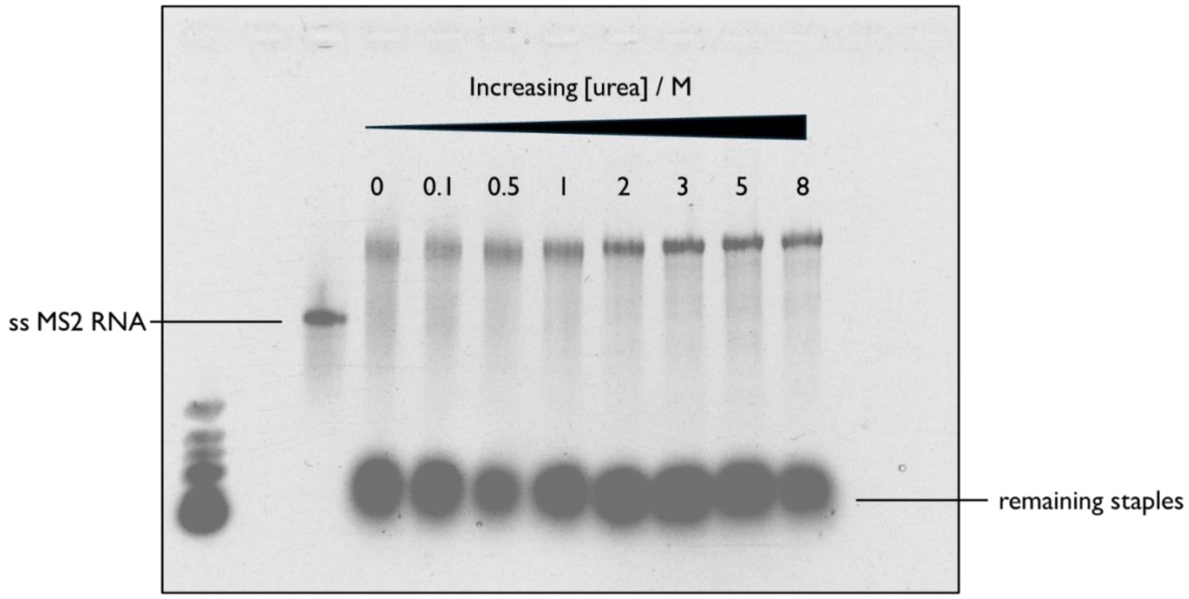

Figure S3. Electrophoretic analysis of DNA-duplexed MS2 RNA hybridized with its DNA complments (5x stoichiometric excess) in the presence of increasing concentrations of urea at 25 °C for 12 h. Lanes: L = Low range DNA ladder (New England Biolabs). Single-stranded MS2 RNA directly from freezer storage is included for comparison. The main band corresponds to the duplexed species. An excess of DNA staples can be observed at the bottom of the gel, which was run prior to Amicon filtration.

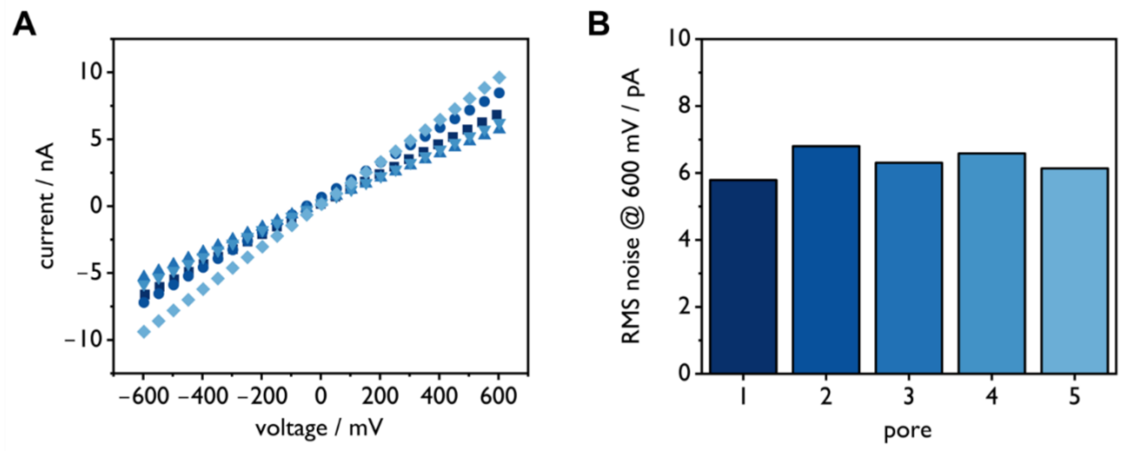

Figure S4. Nanopore characterization data for five representative nanopores used to study the RNA IDs presented in this work. (A) Current-voltage curves, depicting linear behaviour. (B) Root-mean-square noise at an applied voltage of V = 600 mV for the five pores. Pores with an RMS noise > 7 pA were not used. A frequency cut off of 50 kHz was employed. All nanopore characterisation was carried out using 4 M LiCl (with 1 x TE) in both the cis and trans reservoirs of the nanopore chip.

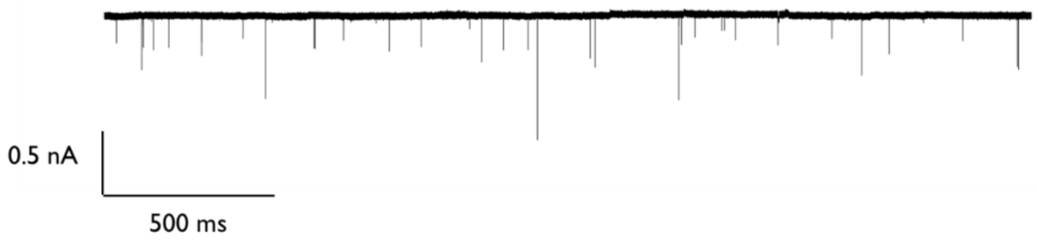

Figure S5. Raw current-time trace for a sample of MS2 RNA ID '1 0 1'. Samples were incubated with the DNA complements at 25 °C for 12 hours with 5 M urea. The concentration for measurement was 1 nM (as measured by NanoDrop spectrophotometer) of RNA:DNA duplex in 4 M LiCl.

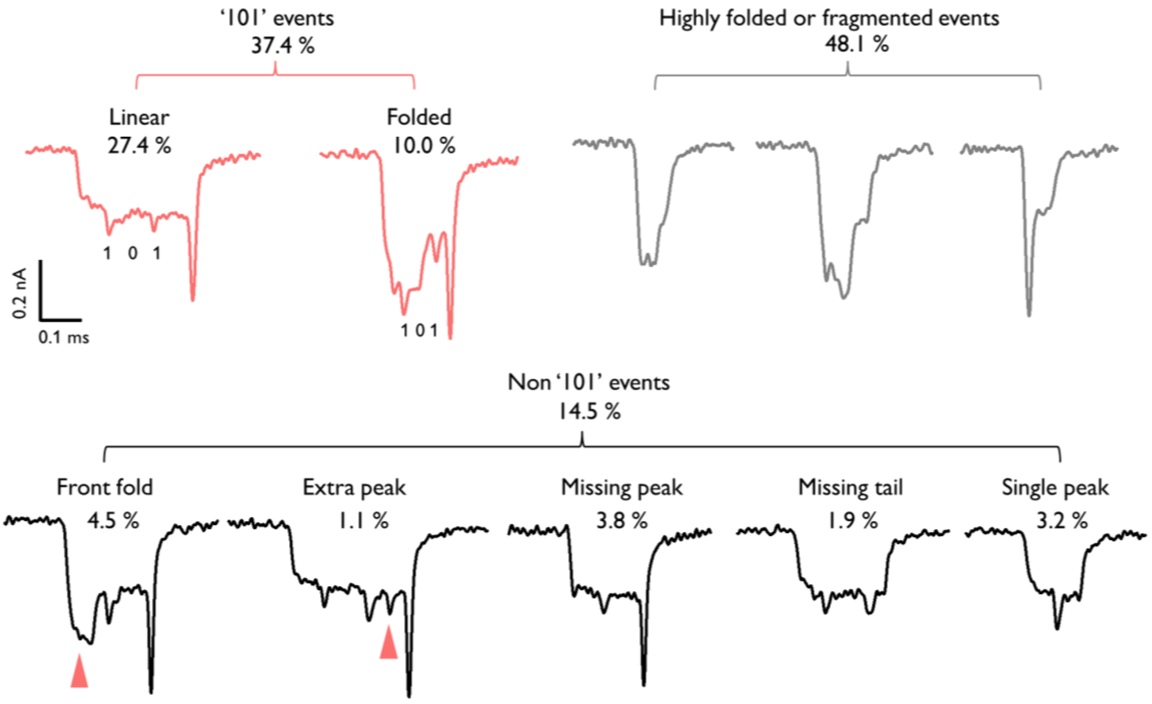

Figure S6. Gallery of different event types and their relative populations in single-nanopore experiment. MS2 "1 0 1" RNA IDs were formed isothermally (T = 25 °C, t = 12 h) in the presence of 5 M urea (100 mM LiCl, 10 mM Tris-HCl, pH 7.5). A total of 1000 nanopore translocations were recorded and the events were categorized by inspecting the number of current levels. 37.4 % of events could be described as "1 0 1" translocations, meaning they conform to the designed barcode, and translocate in a linear or folded conformation. Of these, nearly three quarters are linear (single-file translocation) under these conditions. 48.1% of translocations show no discernible features, given the conformation in which the ID can translocate through the nanopore, which may hinder the localization of the barcode. RNA fragments would also be included here. Finally, we observe translocation events ascribed to the RNA IDs that do not hold the entire '101' barcode. These originate from folds which obscure the barcode (such as a front fold), the presence of knots,[5] fragmentation of IDs, or the combination of any of these within a single molecule. Overall, these are much less numerous than the highly folded events, demonstrating that folding is a more significant hinderance to barcode identification than misassembly.

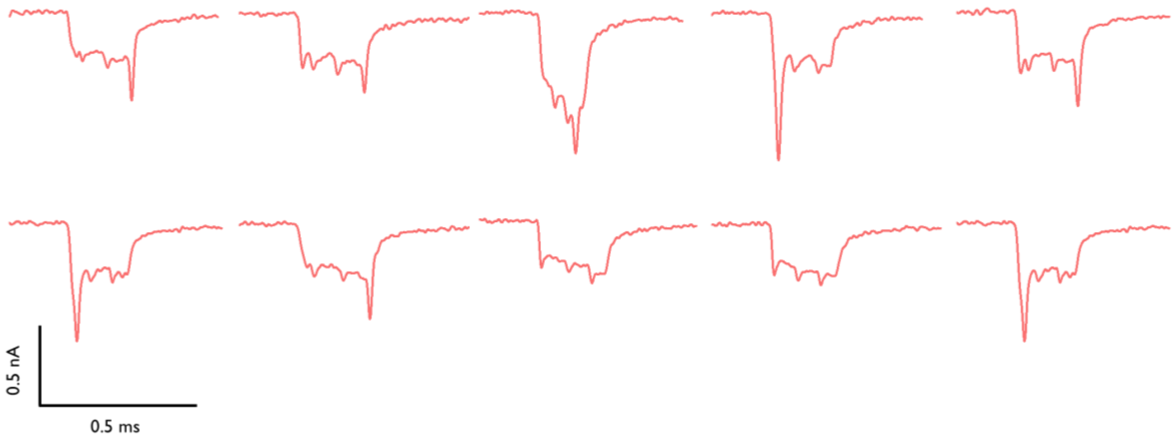

Figure S7. Example nanopore translocation events for MS2 RNA barcode '1 0 1'. Samples were produced by incubating the RNA with its corresponding DNA complements at 25 °C for 12 hours with 5 M urea. The events shown are the first 10 '1 0 1' translocations (folded and unfolded) from the experiment.

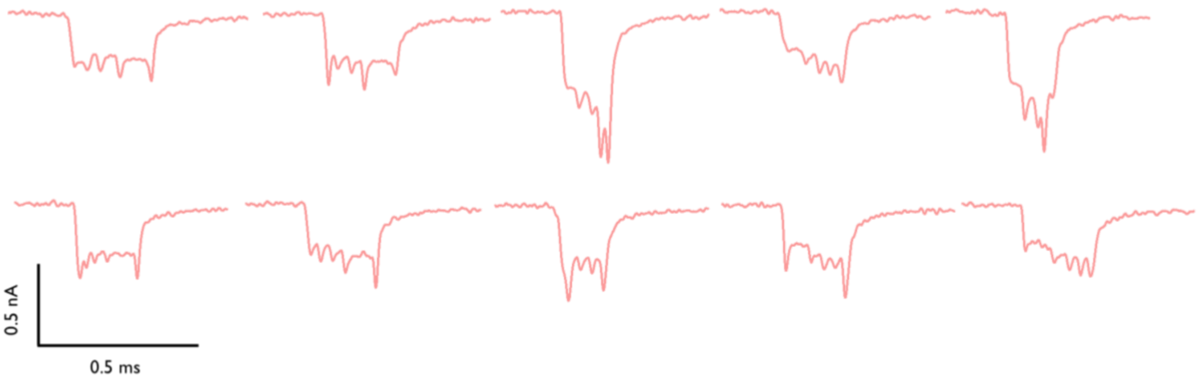

Figure S8. Example nanopore translocation events for MS2 RNA barcode '1 1 1 1'. Samples were produced by annealing the RNA with its corresponding DNA complements from 70 °C to 4 °C over 45 min. The events shown are the first 10 '1 1 1 1' translocations (folded and unfolded) from the experiment.

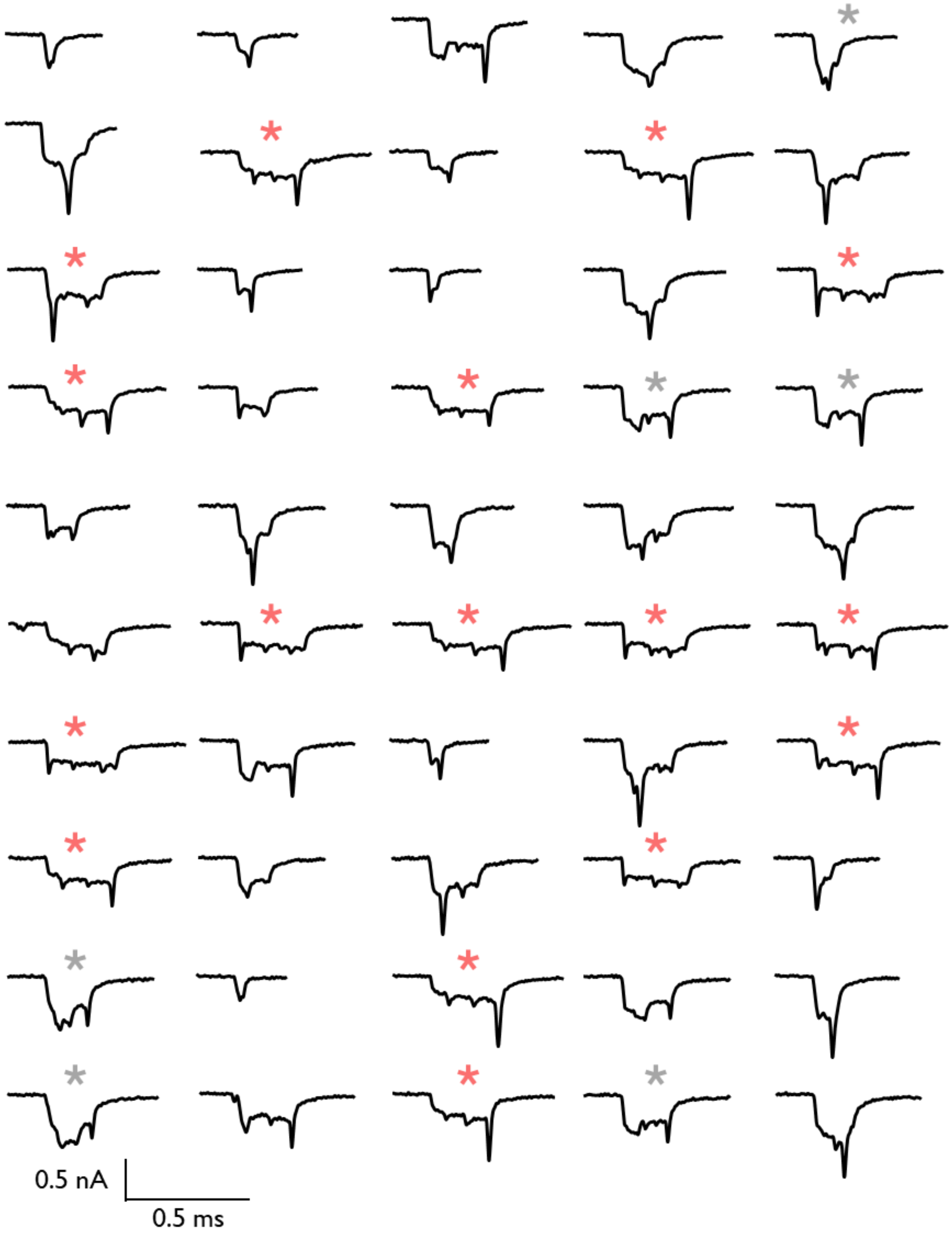

Figure S9. Gallery of first 50 nanopore translocations for the urea-annealed (T = 25 °C, t = 12 h) MS2 RNA. The '1 0 1' barcode was employed. Signal corresponding to '1 0 1' are marked with asterisks, with the orange marking linear (single-file) translocations, while folded events are marked with grey.

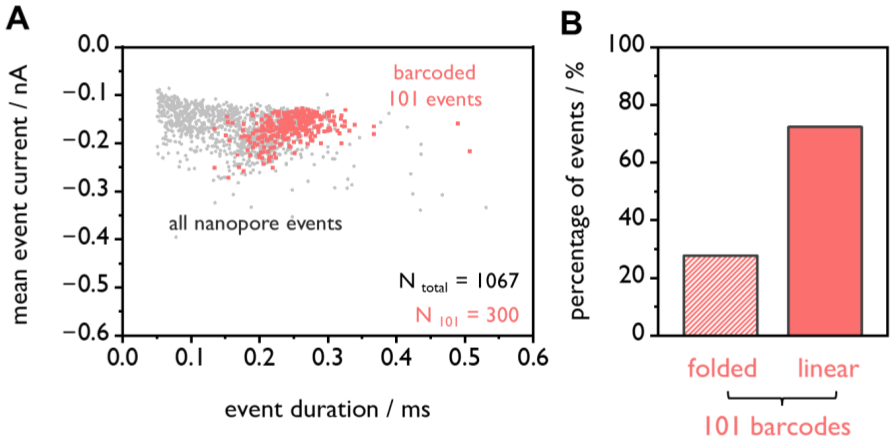

Figure S10. Nanopore data for "1 0 1" MS2 RNA IDs formed isothermally (T = 25 °C, t = 12 h) in the presence of 2.5 M urea (100 mM LiCl, 10 mM Tris-HCl, pH 7.5). (A) Mean event current as a function of event duration. A total of 1067 single-molecule translocations were measured, with 300 traces matching the '1 0 1' barcode design by inspection. (B) Of the 300 '1 0 1' traces, 217 were found to be linear.

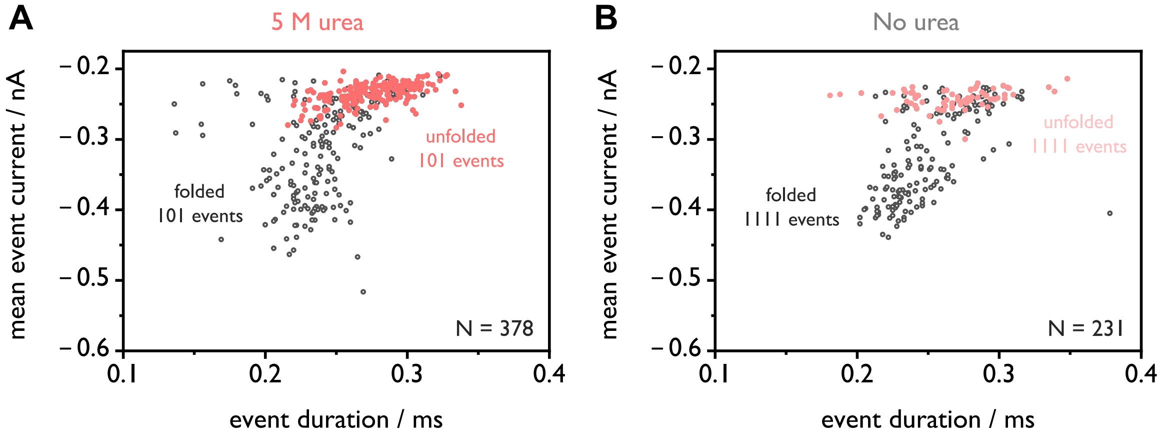

Figure S11. Scatter plots of mean event current as a function of event duration for (A) "1 0 1" RNA IDs formed isothermally in the presence of 5 M urea (T = 25 °C, t = 12 h), and (B) "1 1 1 1" RNA IDs formed via thermal annealing (70 °C to 4 °C over 45 min). Both samples were made in 100 mM LiCl, 10 mM Tris-HCl, pH 7.5. Coloured events are those identified as completely unfolded (linear translocations), while black circles indicate all other barcoded events. Both (A) and (B) are from a single nanopore experiment and thus were measured simultaneously in the same nanopore to account for pore-to-pore variability.

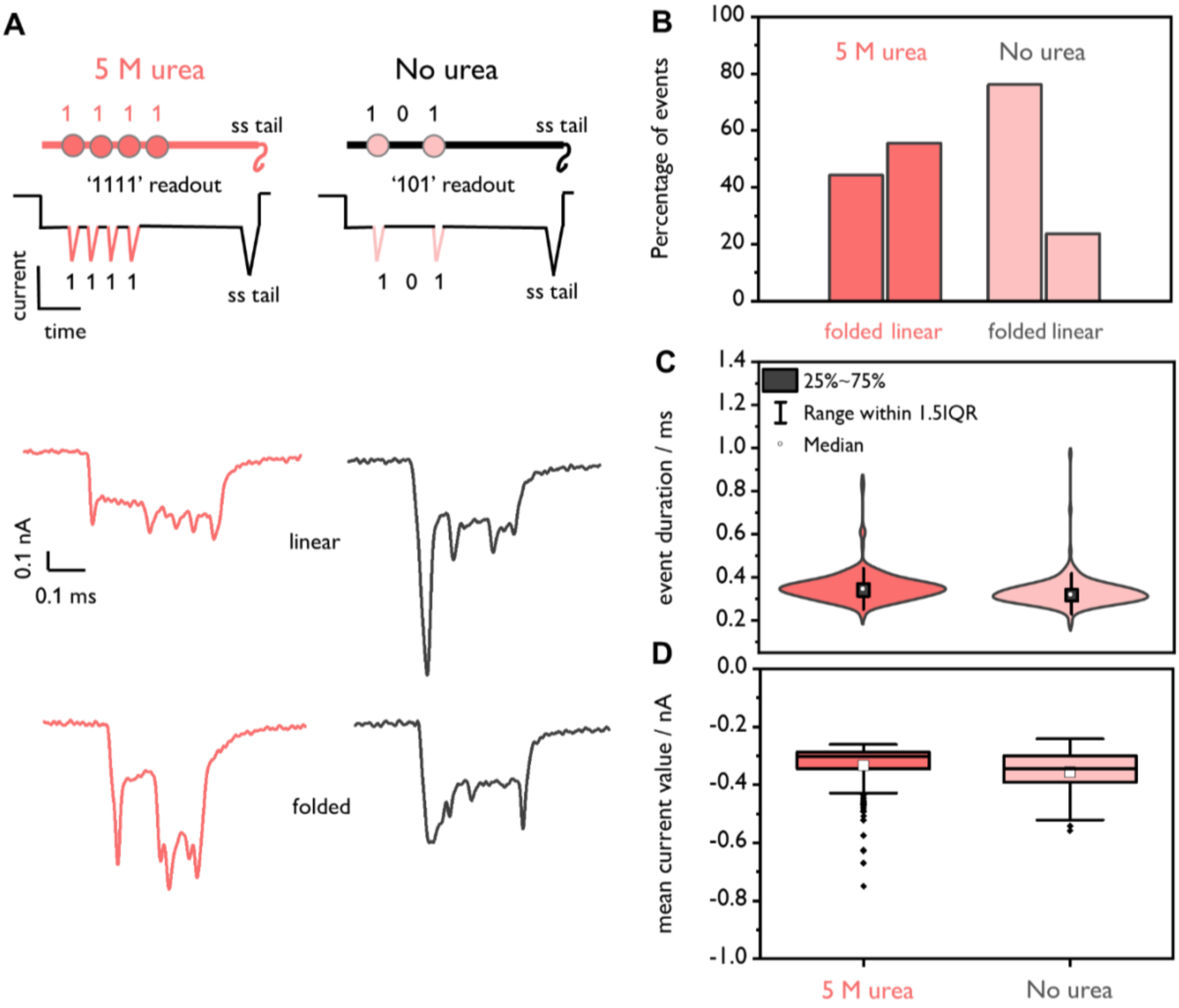

Figure S12. Control experiment wherein barcodes for the with/without urea samples were reversed. "1 1 1 1" RNA IDs were formed isothermally in the presence of 5 M urea (T = 25 °C, t = 12 h), while the "1 0 1" RNA IDs were formed via thermal annealing (70 °C to 4 °C over 45 min). Both samples were made in 100 mM LiCl, 10 mM Tris-HCl, pH 7.5. (A) Schematics of barcodes assembled in 5 M urea and 0 M urea (left and right, respectively) and example current traces. (B) Comparison of the percentage of events that were folded and linear for each barcode. This experiment was run in one nanopore and the total number of single-molecule events was 2000. (C) No difference was observed in the event duration for samples prepared with or without urea. (D) The differences in the resulting translocations for the two different annealing protocols are reflected in the mean current values for a single experiment, wherein the '1111' barcodes (N = 220 identified barcodes), formed in urea, exhibit a less negative mean current value versus when urea is not used (N = 205 identified barcodes). The colored boxes denote the 25 % - 75 % range, the horizontal lines are the medians, the white squares are the means, and black points are outliers.

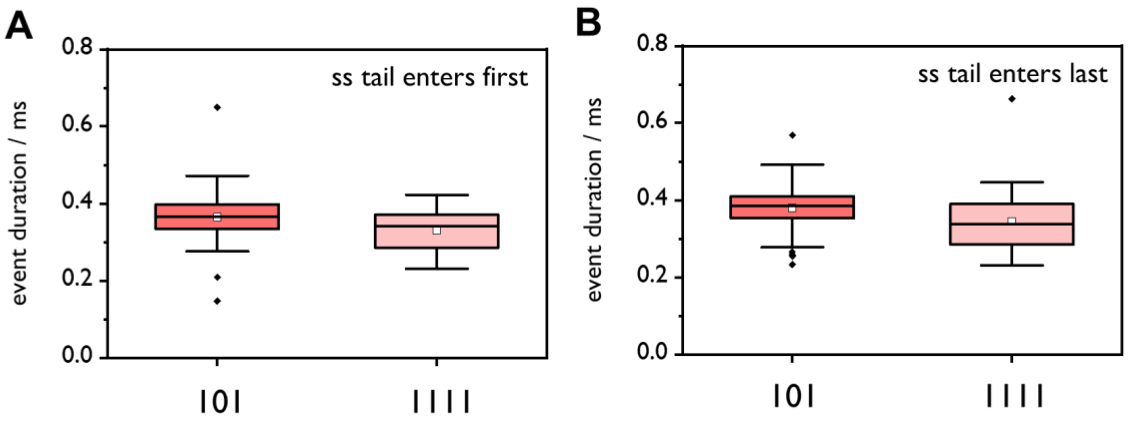

Figure S13. Nanopore event duration is not impacted by the presence of urea, nor by the direction with which the duplex enters the nanopore. The sample is MS2 RNA duplexed with DNA staples. "1 0 1" RNA IDs were formed isothermally (T = 25 °C, t = 12 h) in the presence of 5 M urea while "1 1 1 1" RNA IDs were formed via thermal annealing (ramp from 70 °C to 4 °C over 45 minutes). Both hybridization mixtures contained 100 mM LiCl, 10 mM Tris-HCl, pH 7.5. The colored boxes denote the 25 % - 75 % range, the horizontal lines are the medians, the white squares are the means, and black points are outliers.

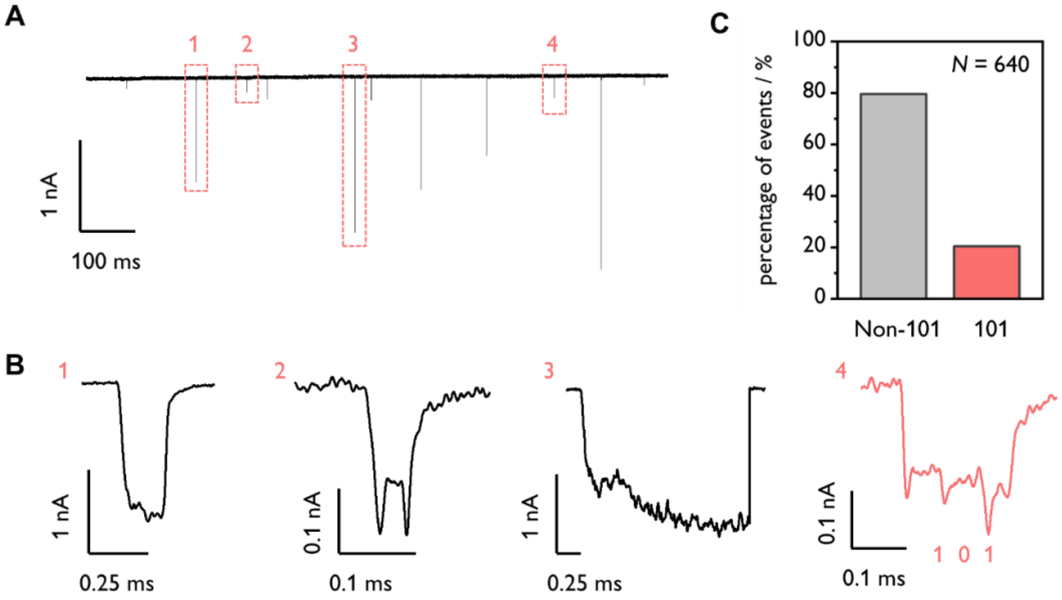

Figure S14. Nanopore data for '1 0 1' barcoded MS2 RNA IDs in total human RNA. The MS2 (10 nM) was spiked into total human RNA (100 nM) and incubated isothermally (T = 25 °C, t = 12 h) in the presence of 5 M urea (100 mM LiCl, 10 mM Tris-HCl, pH 7.5) and DNA staples (50 nM) to form the '1 0 1' barcode. (A) Sample events collated to show the relative peak currents of the different RNA species in this mixed sample. As shown in (B), the smaller peaks such as (4) may display the '1 0 1' label, corresponding to MS2 RNA ID. Much larger peaks, such as (1) and (3) appear to be much larger RNA molecules – possibly the highly abundant ribosomal RNA. (C) Of the total 640 events recorded, 20.4% (131 events) bore the '1 0 1' labelling. While the MS2 was initially only at a ratio of 1:10 relative to the total RNA, it is likely enriched due to degradation/self-cleavage of the single-stranded RNA during room temperature incubation in 100 mM salt.

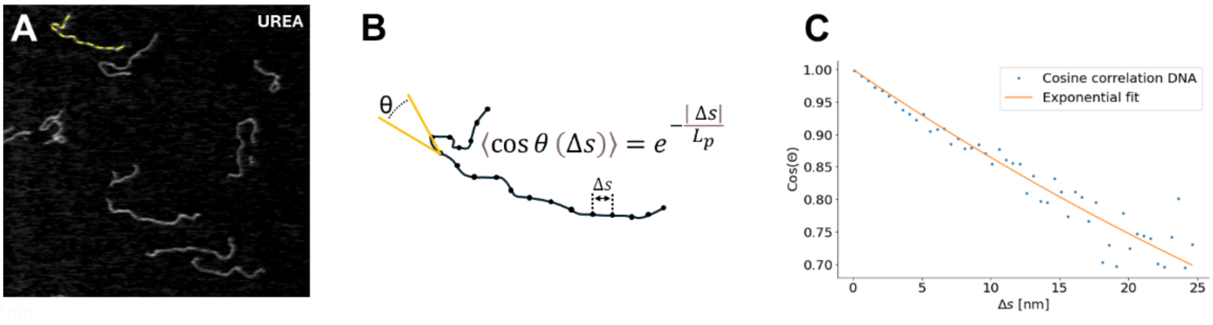

Figure S15. Graphical explanation of the Tangent vector autocorrelation fitting explained in Supplementary Section 7. The DNA strands are traced using the NeuronJ plugin in ImageJ (A). The angle difference is calculated between 2 adjacent edges separated by Δs in contour length (B). The results are then fitted to the exponential function of the persistence length (C).

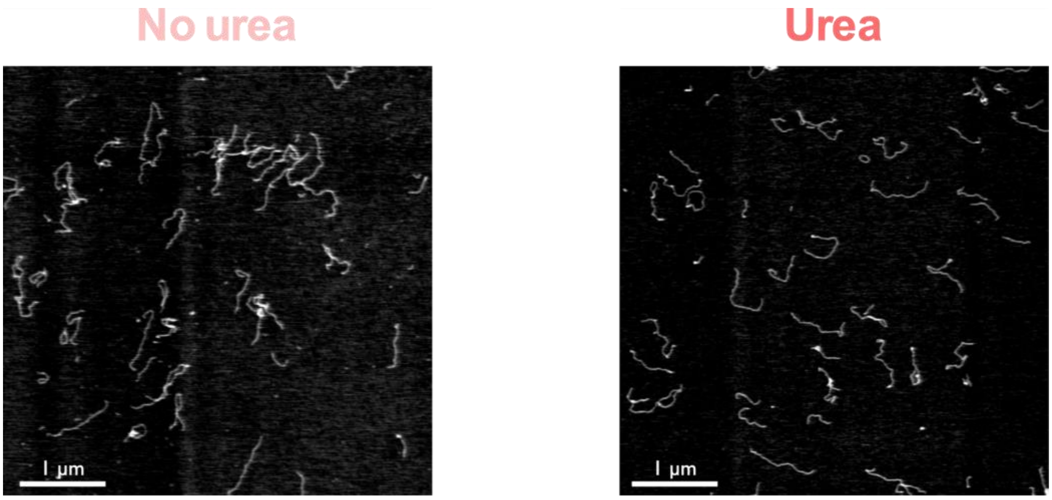

Figure S16. Wider view of the atomic force microscopy of MS2 RNA duplexed with DNA staples with and without urea. The "Urea" RNA IDs were formed isothermally (T = 25 °C, t = 12 h) in the presence of 5 M urea while the "No urea" RNA IDs were formed via thermal annealing (ramp from 70 °C to 4 °C over 45 minutes). Both hybridization mixtures contained 100 mM LiCl, 10 mM Tris-HCl, pH 7.5.

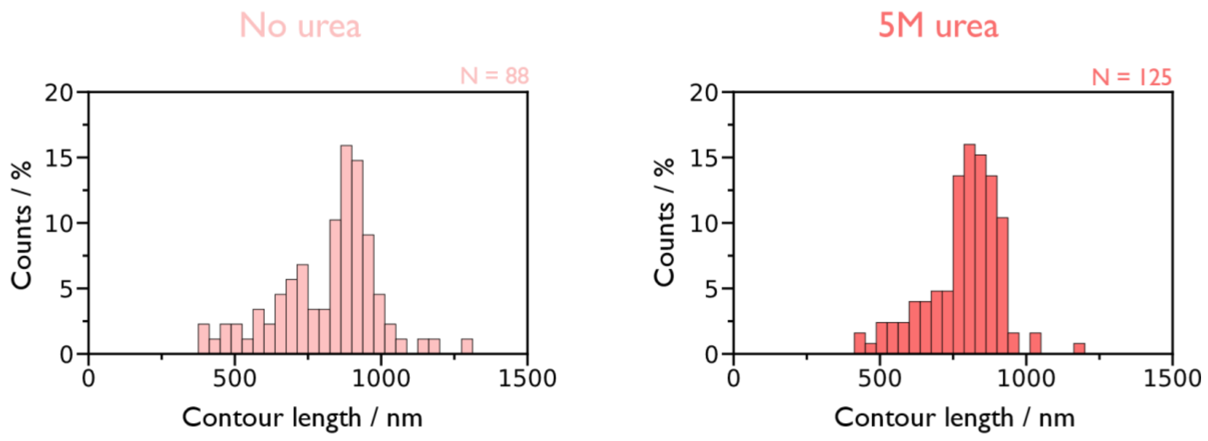

Figure S17. Contour lengths of the RNA:DNA hybrids used for estimating the persistence length from the AFM data. The "Urea" RNA IDs were formed isothermally (T = 25 °C, t = 12 h) in the presence of 5 M urea while the "No urea" RNA IDs were formed via thermal annealing (ramp from 70 °C to 4 °C over 45 minutes). Both hybridization mixtures contained 100 mM LiCl, 10 mM Tris-HCl, pH 7.5.

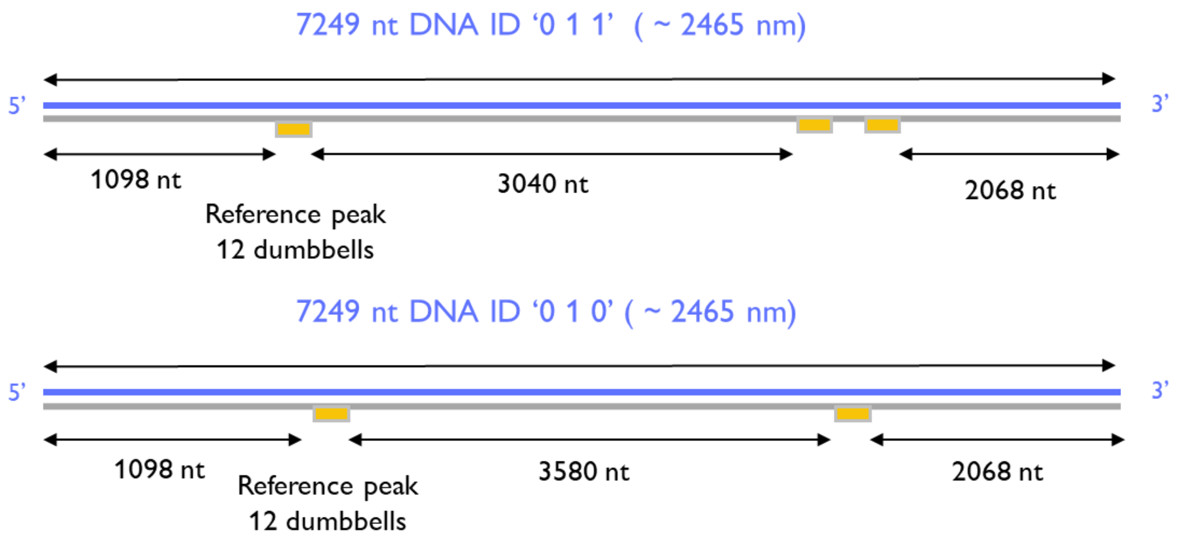

Figure S18. Designs for DNA barcodes. Approximate lengths for the constructs are given assuming a B-form helical geometry.

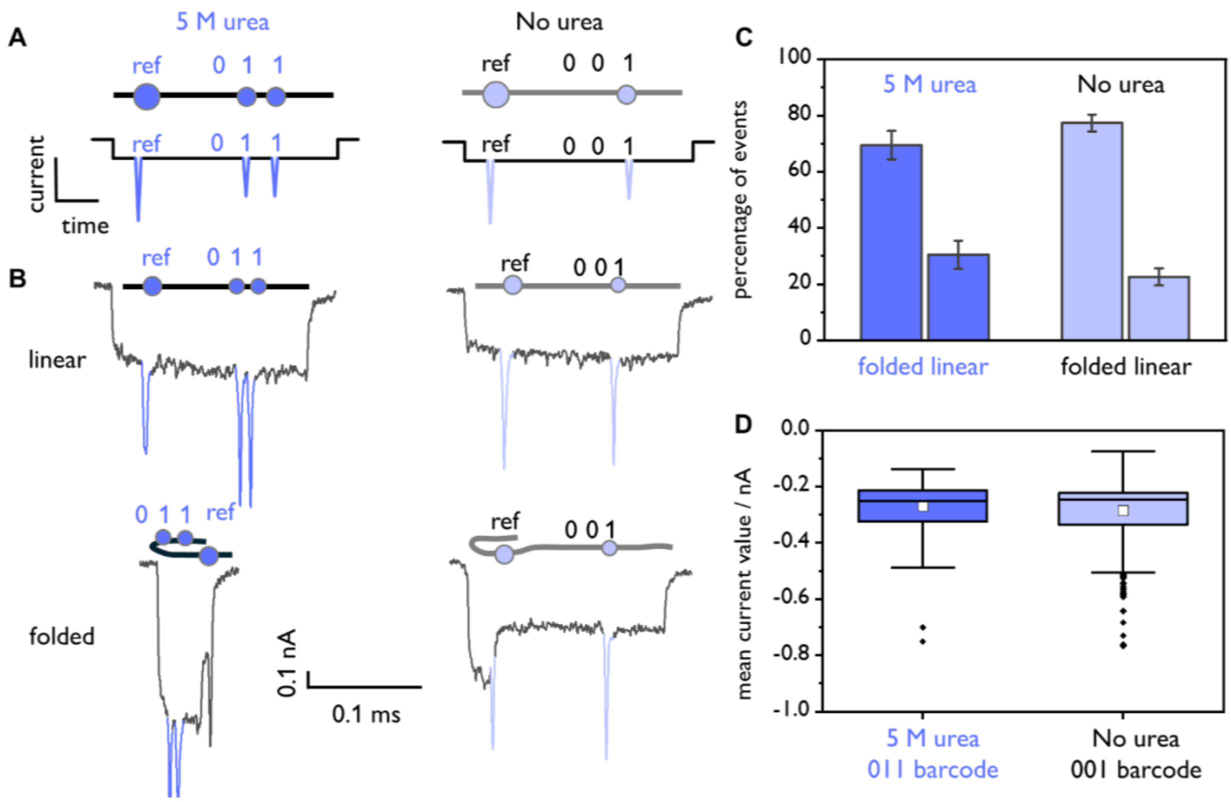

Figure S19. Solid-state nanopore data for DNA duplexes annealed with and without urea. "0 1 1" DNA IDs were formed isothermally (T = 25 °C, t = 12 h) in the presence of 5 M urea while "0 0 1" DNA IDs were formed via thermal annealing (ramp from 70 °C to 4 °C over 45 minutes). Both hybridization mixtures contained 100 mM LiCl, 10 mM Tris-HCl, pH 7.5. The with and without urea samples are then combined (equimolar ratio) in measurement buffer and introduced to the same nanopore. (A) Predicted nanopore current-time trajectories for the '011' and '001' DNA IDs based on the barcode designs. Both barcodes feature a reference (ref) peak to indicate directionality. (B) Real nanopore events for the '011' DNA ID prepared in the presence of 5 M urea and the '001' DNA ID, annealed without urea. Example events are shown for both the linear and folded conformations. (C) When urea is employed in the folding procedure, the number of linear events remains constant (within the error). The error bars represent the standard deviation between 3 measurements made in different nanopores, to account for pore-to-pore variability. (D) The differences in the resulting translocations for the two different annealing protocols are reflected in the mean current values (in a single measurement), wherein the '011' barcodes (N = 567) are formed in urea and the '001' barcodes (N = 742) are formed without. The colored boxes denote the 25 % - 75 % range, the horizontal lines are the medians, the white squares are the means, and black points are outliers.

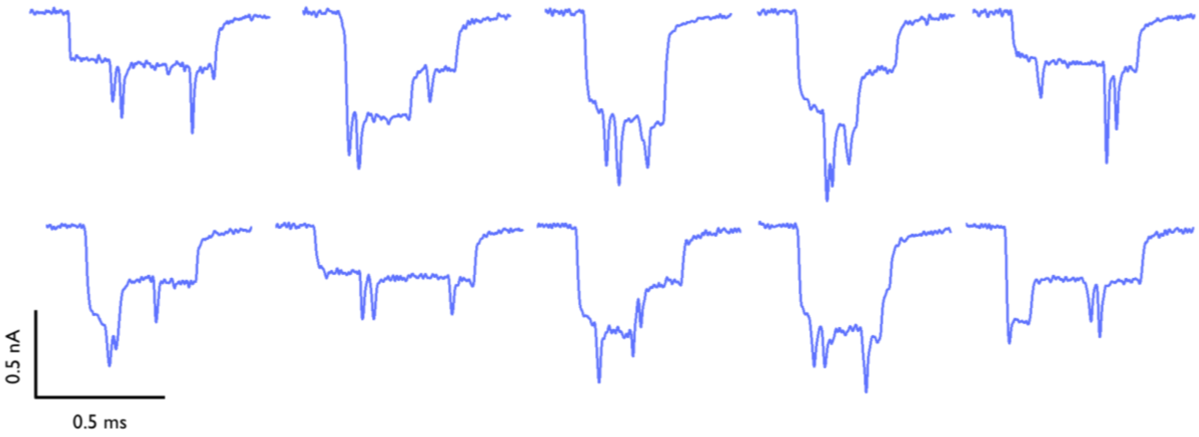

Figure S20. Example nanopore translocation events for M13 DNA barcode '0 1 1'. Samples were incubated at 25 °C for 12 hours with 5 M urea (100 mM LiCl, 10 mM Tris-HCl, pH 7.5). The events shown are the first 10 '0 1 1' translocations (folded and unfolded) from the experiment.

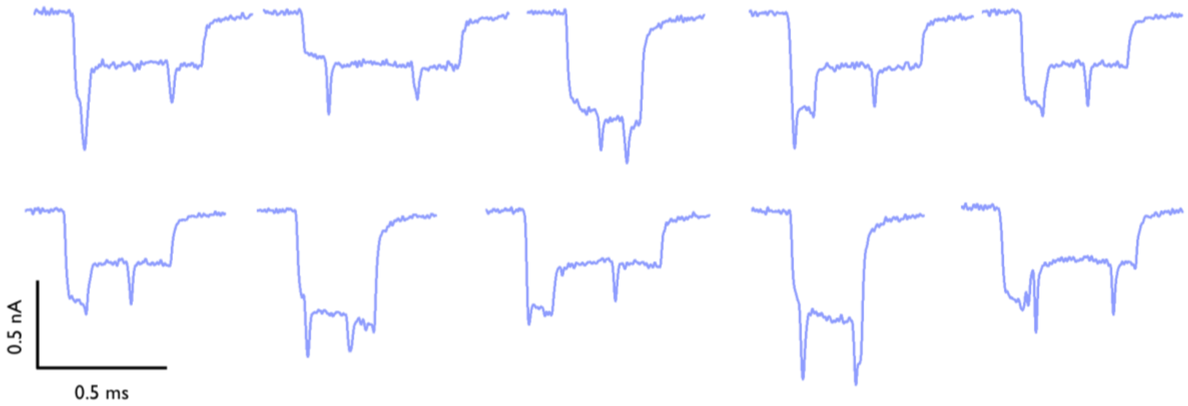

Figure S21. Example nanopore translocation events for M13 DNA barcode '0 0 1'. Samples were annealed from 70 °C to 4 °C over 45 min (100 mM LiCl, 10 mM Tris-HCl, pH 7.5). The events shown are the first 10 '0 0 1' translocations (folded and unfolded) from the experiment.

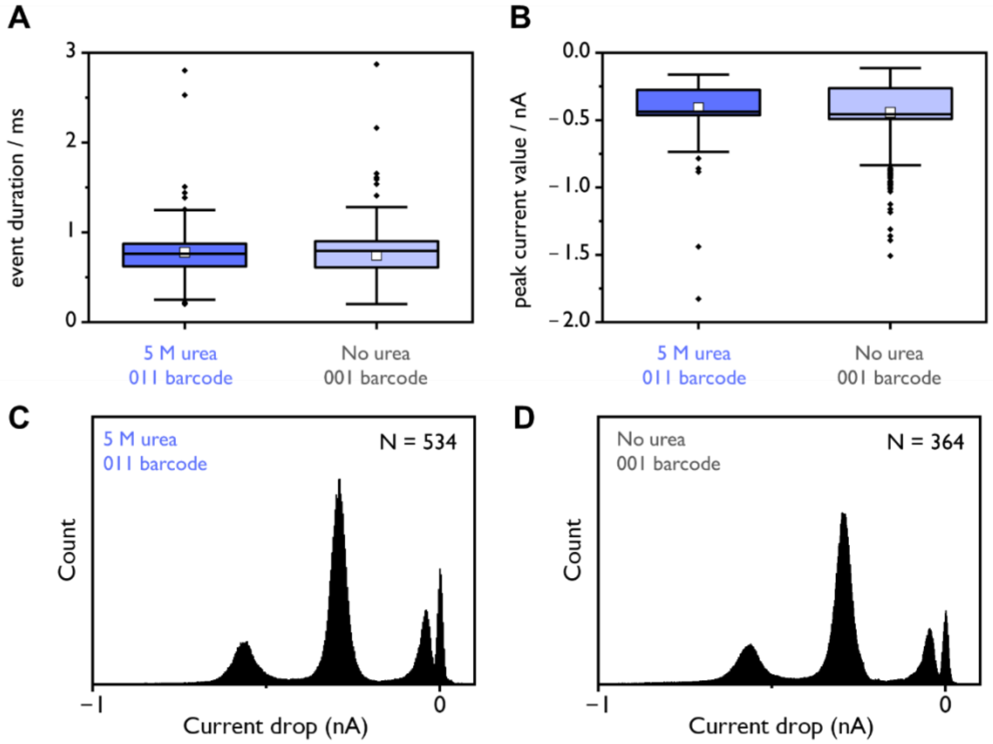

Figure S22. (A) Event duration and (B) peak current nanopore data for M13 DNA IDs with '011' and '001' barcodes. The '011' barcode was formed at T = 25 °C for 12 hours in the presence of 5 M urea (100 mM LiCl, 10 mM Tris-HCl, pH 7.5), while the '001' barcode was formed by annealing from 70 °C to 4 °C over 45 minutes (100 mM LiCl, 10 mM Tris-HCl, pH 7.5). The measurements were made by forming each barcode separately, then combining the samples in an equimolar ratio. The combined sample was then introduced to the glass nanopore. (C) and (D) represent the all-points histograms for the current levels observed for the chemically annealed and thermally annealed samples, respectively. The populations of the different current levels are equal, indicating there is no difference in folding between these two sample preparation methods for DNA:DNA duplexes.

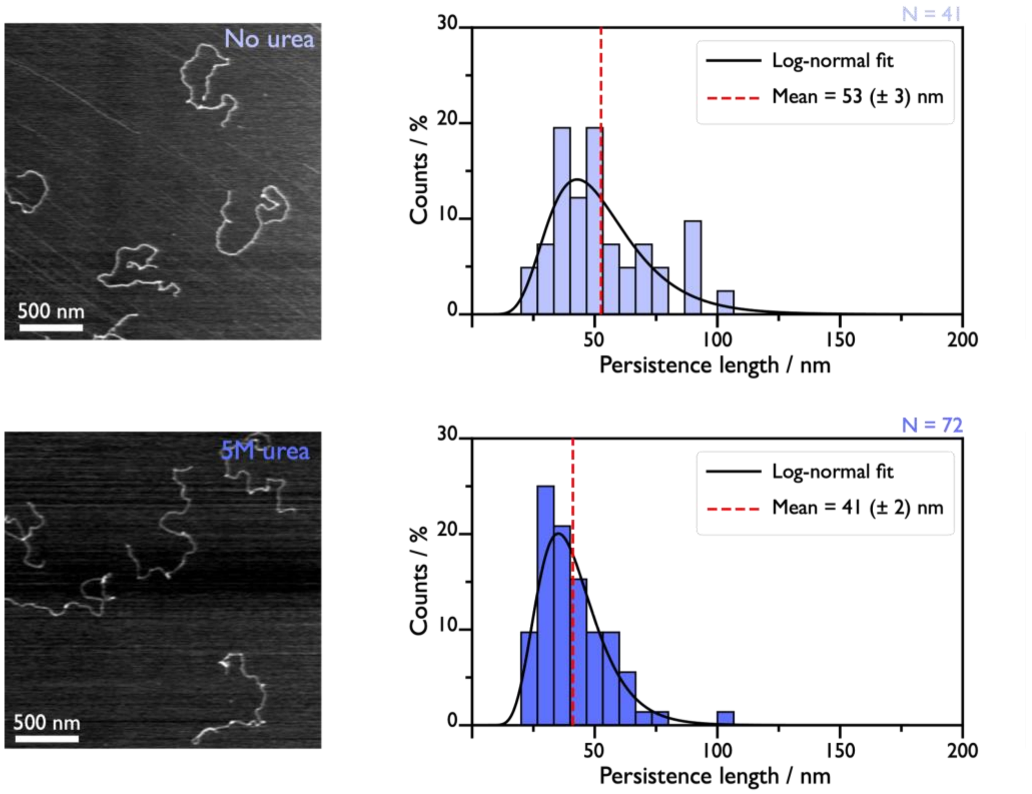

Figure S23. Atomic force microscopy of M13 DNA duplexed with DNA staples in the absence (A) and presence (B) of 5 M urea. The "Urea" DNA IDs were formed isothermally (T = 25 °C, t = 12 h) in the presence of 5 M urea while the "No urea" DNA IDs were formed via thermal annealing (ramp from 70 °C to 4 °C over 45 minutes). Both hybridization mixtures contained 100 mM LiCl, 10 mM Tris-HCl, pH 7.5. Scale bar = 500 nm. Tangent vector autocorrelation fitting reveals the difference in persistence length. Fitting parameters are detailed in the Supplementary Section 7.

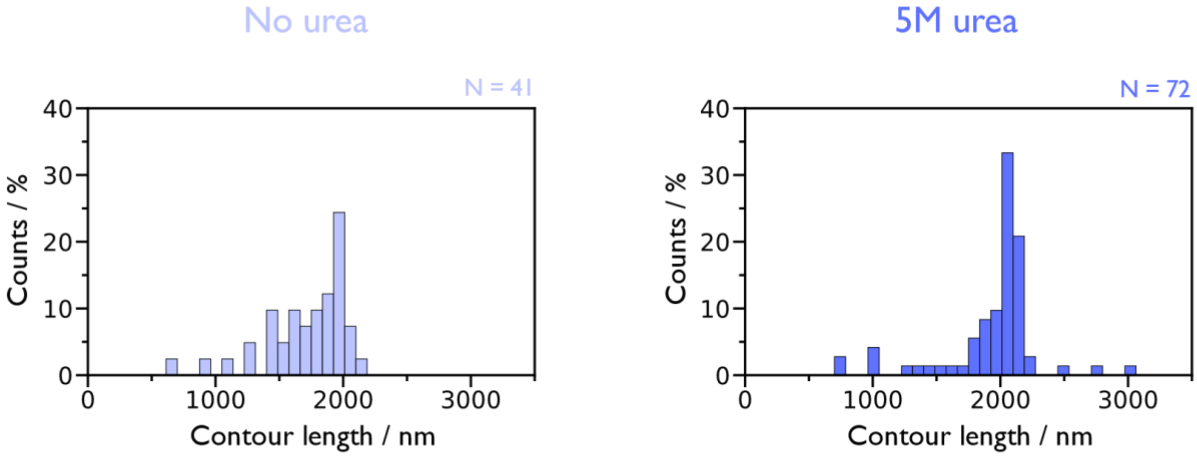

Figure S24. Contour lengths of the DNA:DNA hybrids used for estimating the persistence length from the AFM data. The "Urea" DNA IDs were formed isothermally (T = 25 °C, t = 12 h) in the presence of 5 M urea while the "No urea" DNA IDs were formed via thermal annealing (ramp from 70 °C to 4 °C over 45 minutes). Both hybridization mixtures contained 100 mM LiCl, 10 mM Tris-HCl, pH 7.5.

10. Oligonucleotide sequences

Table S1. Oligonucleotide sequences for DNA strands complementary to MS2 RNA and producing the 101 RNA barcode. Dumbbell-containing sequences are highlighted in red.

NAME	SEQUENCE (5' TO 3')	LENGTH (NT)
MS2_1010_1	GGCATTAGCTCGACAGGAAGTTGAGCAGGACCCCGAAAGGGGTCCCACCC	50
MS2_1010_2	GCTACCTACAGCGATAGCCATGGTAGCGTCTCGCTAAAGACATTAAAAAT	50
MS2_1010_3	AAGGGTACTAAAAGCTCGCACAGGTCAAACCTCCTAGGAATGGAATTCCG	50
MS2_1010_4	CCGTCCGCGTAAACGCGAACGGAGGGGACGAAGGTCTCGTTCTCCCTATC	50
MS2_1010_5	GAGTCCAGTTCGAACGATATTTTAAAGAGAATGAGTTATCTTCAGTCTCA	50
MS2_1010_6	GGGGTAGTGCCACTGTTTCGTTTTGGCCCCAGTCGAGTTAAAACGACCGG	50
MS2_1010_7	GCACCTTGATCTATCGATGTGACACTTAACGCCCCCCGTGAATACGGAGA	50
MS2_1010_8	CCGGCTTTCTCCTCGTACGGGCGACCCCACGATGACCCACTTCGCTTGTAG	51
MS2_1010_9	CACTCCGTTCCCTACAACGAGCCTAAATTCATATGACT	38
MS2_1010_10	CGTTATAGCGGACCGCGTGTCTGATCCACGGCGCACAT	38
MS2_1010_11	TGGTCTCGGACCAATAGAGCCGCTCTCAGAGCGCGGGG	38
MS2_1010_12	GGTAACGGTTGCTTGTTCAGCGAACTTCTTGTAAGGCG	38
MS2_1010_13	CTGCATCCTGCAACTTGTGCCCCATAGGAGCACCGTTG	38
MS2_1010_14	GAGAACGTGCATTGCCCAAACAACGACGATCGGTAGCC	38
MS2_1010_15	AGAGAGGAGGTTGCCAATAAGGCTACGGATGCTGGTTT	38
MS2_1010_16	GTAAAACATCCGGATCCCATGACAAGGATTTGTCATGT	38
MS2_1010_17	AAGAAACCTTCTCTATTTATCTGACCGCGATCACCATT	38
MS2_1010_18	CGCCTCCCGTTCCTCTTTTGAGGAACAAGTTTTCTTGTAGCTTAGCGA	48
MS2_1010_19	TAGCTAAGGTTCCTCTTTTGAGGAACAAGTTTTCTTGTACGACGGGTC	48
MS2_1010_20	GCCTCGTCATTCCTCTTTTGAGGAACAAGTTTTCTTGTTACCAGAACC	48
MS2_1010_21	TAAGGTCGGATCCTCTTTTGAGGAACAAGTTTTCTTGTTGCTTTGTGA	48
MS2_1010_22	GCAATTCGTCTCCTCTTTTGAGGAACAAGTTTTCTTGTCCTTAAGTAA	48
MS2_1010_23	GCAATTGCTGTCCTCTTTTGAGGAACAAGTTTTCTTGTTAAAGTCGTC	48
MS2_1010_24	ACTGTGCGGATCACCGCTTCCAGTAGCGACAG	32
MS2_1010_25	AAGCAATTGATTGGTAAATTTCGAGAGAAAGATCGCGA	38
MS2_1010_26	GGAAGATCAATACATAAAGAGTTGAACTTCTTTGTTGT	38
MS2_1010_27	CTTCGACATGGGTAATCCTCATGTTTGAATGGCCGGCG	38
MS2_1010_28	TCTATTAGTAGATGCCGGAGTTTGCTGCGATTGCTGAG	38
MS2_1010_29	GGAATCGGGTTTCCATCTTTTAGGAGACCTTGCATTGC	38
MS2_1010_30	CTTAACAATAAGCTCGCAGTCGGAATTCGTAGCGAAAA	38
MS2_1010_31	TTGGAATGGTTAGTTCCATATTTAAGTACGAACGCCAT	38
MS2_1010_32	GCGGCTACAGGAAGCTCTACACCACCAACAGTCTGGGT	38
MS2_1010_33	TGCCACTTTAGGCACCTCGACTTTGATGGTGTATTTGC	38
MS2_1010_34	GATTCTGCGCAGAGCTCTGACGAACGCTACAGGTTACT	38
MS2_1010_35	TTGTAAGCCTGTGAACGCGAGTTAGAGCTGATCCATTC	38
MS2_1010_36	AGCGACCCCGTTAGCGAAGTTGCTTGGGGCGACAGTCA	38
MS2_1010_37	CGTCGCCAGTTCCGCCATTG	20
MS2_1010_38	TCGACGAGAACGAACTGAGT	20
MS2_1010_39	AAAGTTAGAAGCCATGCTTC	20
MS2_1010_40	AAACTCCGGTTGAGGGCTCT	20

MS2_1010_41	ATCTAGAGAGCCGTTGCCTG	20
MS2_1010_42	ATTAATGCTAACGCATCTAA	20
MS2_1010_43	GGTATGGACCATCGAGAAAGGAGACTTTACGT	32
MS2_1010_44	ACGCGCCAGTTGTTGGCCATACGGATTGTACCCCTCGA	38
MS2_1010_45	TGCATGGCTGAGATTTGGGCCTTAGCAGTGCCCTGTCT	38
MS2_1010_46	CTCCACAGTCCACCCGTAGGGAGCGTCAACGCTTATGA	38
MS2_1010_47	TGGACTCACCCGTTATTACGTCAGTAACTGTTCCTGAC	38
MS2_1010_48	ATGTAGGAGCATCCCACGGGGGCCGTAAGGCCCTCGAG	38
MS2_1010_49	CATGTTACCTACAGGTAGGAGCCAGTCGACAACGAATG	38
MS2_1010_50	AGAAAGGCACCTTTTCCCACACTATACCTAGTGGGTTC	38
MS2_1010_51	AAGATACCTAGAGACGACAACCATGCCAAACGTGCATC	38
MS2_1010_52	GTTTATGTAAAACCATATCACGATACGTCGCGATATGT	38
MS2_1010_53	TGCACGTTGTTCCTCTTTTGAGGAACAAGTTTTCTTGTCTGGAAGTTT	48
MS2_1010_54	GCAGCTGGATTCCTCTTTTGAGGAACAAGTTTTCTTGTACGACAGACG	48
MS2_1010_55	GCCATCTAACTCCTCTTTTGAGGAACAAGTTTTCTTGTTTGATGTTAG	48
MS2_1010_56	TACCGACCTGTCCTCTTTTGAGGAACAAGTTTTCTTGTACGTACGGCT	48
MS2_1010_57	CTCATAGGAATCCTCTTTTGAGGAACAAGTTTTCTTGTGAAACTCTTG	48
MS2_1010_58	AAGGTGAACCTCCTCTTTTGAGGAACAAGTTTTCTTGTTTCGTAAGCA	48
MS2_1010_59	TCTCATATGCACCCTGGATATCACTCATTAGT	32
MS2_1010_60	GGTAACCAACCGAACTGCAACTCCAACCACCTGCCGGC	38
MS2_1010_61	CACGTGTTTTGATCGAAACTTTCGATCTTCGTTTAGGG	38
MS2_1010_62	CAAGGTAGCGGAGCGCCTGGCGCCAATTACCGCGACGA	38
MS2_1010_63	GCGGCAGTGTACGCCTTCACGAGCGCAATGGTTTGCGT	38
MS2_1010_64	CGCGAGTTGTGAGGCTGTCGACCTGGCCTCTGCTAAAG	38
MS2_1010_65	CAACACCAAGGTTAAAATTACCCTGGGTGACCTTTTGC	38
MS2_1010_66	AGGACTTCGGTCGACGCCCGGTTCGCAACGTTCTGCGG	38
MS2_1010_67	CACTTCGATGTAAGTCAAGTTTTGGCTTACAGGGAAGA	38
MS2_1010_68	GGCTGTAGCAGGAGCGTGCGTCGAGGGAGAAGCCGAAA	38
MS2_1010_69	AGGCAGCCCGATCTATTTTATTATTCTTCGGAACTGTAAA	40
MS2_1010_70	ACCCCTTTCTGGAGGTACATATTCATATCAGGCTCCTTAC	40
MS2_1010_71	CATTCAGGTCTATACCAACGGATTTGAGCCGGCGTCTGATGAAAGCACCG	50
MS2_1010_72	CGAACCATCTACGCTGCCCTGCTGAGCCAGACGCTGGTTGATCGATTGAT	50
MS2_1010_73	AGGCGATCGGAGATGGAATCGGATGCAGACGATAAGTCTATCGTCGCAAG	50
MS2_1010_74	GGATACGATCGAGATATGAATATAGCTCTGGTGGGAGAAAACTCCACACC	50
MS2_1010_75	AAATAGTTCCCATCGTATCGTCTCGCCATCTACGATTCCGTAGTGTGAGC	50
MS2_1010_76	CAGAATATCATGGACTCTAGCTCAAATGTGAACCCATTTCCCATTGTGGA	50
MS2_1010_77	CTATGGTTCCGGCGTTACCAAAATGGATTTGGGTCGCTTTGACTATTGCC	50
MS2_1010_78	CACACGGGGTGCAATCTCACTGGGACATATAATATCGTCCCCGTAGATGC	50
MS2_1010_79	AACGTTTTACGAAGATTCGGTTTAAAACCGTAGTAGGCAAGTGCCTCTAG	50
MS2_1010_80	CACCACGGTAAAAGTGCGCGCCGCAGCTCTCGCGAAAGAGCCCGGACACG	50
MS2_1010_81	GGCGAAGAGATTGTCAACAGGTTTCTTGATGTAAAACGGTTTGACATCGA	50
MS2_1010_82	ATACCTCCGACAACTCCCCAACCCCGTAGCCGATTTAATATCAGCATCAG	50
MS2_1010_83	GCACCTGGGAGGAGAGCCGTACCCACACCTTATAGAGGCGTGGATCTGAC	50
MS2_1010_84	GACTACGTAGTAGTCGGCAGCGAGGTCCGTCCCACCGAAGAACATCGAAG	50
MS2_1010_85	AGCCGCCCGTACGGAGTCTTGGTGTATACCGAGACTGCCGTAGGCGGGCT	50
MS2_1010_86	CTCGAGCGATACGAGCAAGACGGAAACCCGAGGTACGGGTATCCGCGAGC	50

MS2_1010_87	CCACGCTATGTAGCGACCACTGTCGTGCTTTTCGCTGAAGAACTTGCGTT	50

Table S2. Oligonucleotide sequences for DNA strands complementary to MS2 RNA and producing the 1111 RNA barcode. Dumbbell-containing sequences are highlighted in red.

NAME	SEQUENCE (5' TO 3')	LENGTH
MS2-BARCODE1111-1	GCTACCTACAGCGATAGCCATGGTAGCGTCTCGCTAAAGACATTAAAAAT	50
MS2-BARCODE1111-2	AAGGGTACTAAAAGCTCGCACAGGTCAAACCTCCTAGGAATGGAATTCCG	50
MS2-BARCODE1111-3	CCGTCCGCGTAAACGCGAACGGAGGGGACGAAGGTCTCGTTCTCCCTATC	50
MS2-BARCODE1111-4	GAGTCCAGTTCGAACGATATTTTAAAGAGAATGAGTTATCTTCAGTCTCA	50
MS2-BARCODE1111-5	GGGGTAGTGCCACTGTTTCGTTTTGGCCCCA	32
MS2-BARCODE1111-6	GTCGAGTTAATCCTCTTTTGAGGAACAAGTTTTCTTGTAACGACCGGG	48
MS2-BARCODE1111-7	CACCTTGATCTCCTCTTTTGAGGAACAAGTTTTCTTGTTATCGATGTG	49
MS2-BARCODE1111-8	ACACTTAACGTCCTCTTTTGAGGAACAAGTTTTCTTGTCCCCCCGTGA	48
MS2-BARCODE1111-9	ATACGGAGACTCCTCTTTTGAGGAACAAGTTTTCTTGTCGGCTTTCTC	48
MS2-BARCODE1111-10	CTCGTACGGGTCCTCTTTTGAGGAACAAGTTTTCTTGTCGACCCCACG	48
MS2-BARCODE1111-11	ATGACCCACTTCCTCTTTTGAGGAACAAGTTTTCTTGTTCGCTTGTAG	48
MS2-BARCODE1111-12	CACTCCGTTCCCTACAACGAGCCTAAATTCATATGACT	39
MS2-BARCODE1111-13	CGTTATAGCGGACCGCGTGTCTGATCCACGGCGCACAT	38
MS2-BARCODE1111-14	TGGTCTCGGACCAATAGAGCCGCTCTCAGAGCGCGGGG	38
MS2-BARCODE1111-15	GGTAACGGTTGCTTGTTCAGCGAACTTCTTGTAAGGCG	38
MS2-BARCODE1111-16	CTGCATCCTGCAACTTGTGCCCCATAGGAGCACCGTTG	38
MS2-BARCODE1111-17	GAGAACGTGCATTGCCCAAACAACGACGATCGGTAGCC	38
MS2-BARCODE1111-18	AGAGAGGAGGTTGCCAATAAGGCTACGGATGCTGGTTT	38
MS2-BARCODE1111-19	GTAAAACATCCGGATCCCATGACAAGGATTTGTCATGT	38
MS2-BARCODE1111-20	AAGAAACCTTCTCTATTTATCTGACCGCGATCACCATT	39
MS2-BARCODE1111-21	CGCCTCCCGTTCCTCTTTTGAGGAACAAGTTTTCTTGTAGCTTAGCGA	48
MS2-BARCODE1111-22	TAGCTAAGGTTCCTCTTTTGAGGAACAAGTTTTCTTGTACGACGGGTC	48
MS2-BARCODE1111-23	GCCTCGTCATTCCTCTTTTGAGGAACAAGTTTTCTTGTTACCAGAACC	48
MS2-BARCODE1111-24	TAAGGTCGGATCCTCTTTTGAGGAACAAGTTTTCTTGTTGCTTTGTGA	48
MS2-BARCODE1111-25	GCAATTCGTCTCCTCTTTTGAGGAACAAGTTTTCTTGTCCTTAAGTAA	48
MS2-BARCODE1111-26	GCAATTGCTGTCCTCTTTTGAGGAACAAGTTTTCTTGTTAAAGTCGTC	48
MS2-BARCODE1111-27	ACTGTGCGGATCACCGCTTCCAGTAGCGACAG	32
MS2-BARCODE1111-28	AAGCAATTGATTGGTAAATTTCGAGAGAAAGATCGCGA	38
MS2-BARCODE1111-29	GGAAGATCAATACATAAAGAGTTGAACTTCTTTGTTGT	38
MS2-BARCODE1111-30	CTTCGACATGGGTAATCCTCATGTTTGAATGGCCGGCG	38
MS2-BARCODE1111-31	TCTATTAGTAGATGCCGGAGTTTGCTGCGATTGCTGAG	38
MS2-BARCODE1111-32	GGAATCGGGTTTCCATCTTTTAGGAGACCTTGCATTGC	38
MS2-BARCODE1111-33	CTTAACAATAAGCTCGCAGTCGGAATTCGTAGCGAAAA	38
MS2-BARCODE1111-34	TTGGAATGGTTAGTTCCATATTTAAGTACGAACGCCAT	38
MS2-BARCODE1111-35	GCGGCTACAGGAAGCTCTACACCACCAACAGTCTGGGT	38
MS2-BARCODE1111-36	TGCCACTTTAGGCACCTCGACTTTGATGGTGTATTTGC	38
MS2-BARCODE1111-37	GATTCTGCGCAGAGCTCTGACGAACGCTACAGGTTACT	38
MS2-BARCODE1111-38	TTGTAAGCCTGTGAACGCGAGTTAGAGCTGATCCATTC	38
MS2-BARCODE1111-39	AGCGACCCCGTTAGCGAAGTTGCTTGGGGCGACAGTCA	38

MS2-BARCODE1111-40	CGTCGCCAGTTCCTCTTTTGAGGAACAAGTTTTCTTGTTCCGCCATTG	48
MS2-BARCODE1111-41	TCGACGAGAATCCTCTTTTGAGGAACAAGTTTTCTTGTCGAACTGAGT	48
MS2-BARCODE1111-42	AAAGTTAGAATCCTCTTTTGAGGAACAAGTTTTCTTGTGCCATGCTTC	48
MS2-BARCODE1111-43	AAACTCCGGTTCCTCTTTTGAGGAACAAGTTTTCTTGTTGAGGGCTCT	48
MS2-BARCODE1111-44	ATCTAGAGAGTCCTCTTTTGAGGAACAAGTTTTCTTGTCCGTTGCCTG	48
MS2-BARCODE1111-45	ATTAATGCTATCCTCTTTTGAGGAACAAGTTTTCTTGTACGCATCTAA	48
MS2-BARCODE1111-46	GGTATGGACCATCGAGAAAGGAGACTTTACGT	32
MS2-BARCODE1111-47	ACGCGCCAGTTGTTGGCCATACGGATTGTACCCCTCGA	38
MS2-BARCODE1111-48	TGCATGGCTGAGATTTGGGCCTTAGCAGTGCCCTGTCT	39
MS2-BARCODE1111-49	CTCCACAGTCCACCCGTAGGGAGCGTCAACGCTTATGA	38
MS2-BARCODE1111-50	TGGACTCACCCGTTATTACGTCAGTAACTGTTCCTGAC	38
MS2-BARCODE1111-51	ATGTAGGAGCATCCCACGGGGGCCGTAAGGCCCTCGAG	38
MS2-BARCODE1111-52	CATGTTACCTACAGGTAGGAGCCAGTCGACAACGAATG	38
MS2-BARCODE1111-53	AGAAAGGCACCTTTTCCCACACTATACCTAGTGGGTTC	38
MS2-BARCODE1111-54	AAGATACCTAGAGACGACAACCATGCCAAACGTGCATC	38
MS2-BARCODE1111-55	GTTTATGTAAAACCATATCACGATACGTCGCGATATGT	38
MS2-BARCODE1111-56	TGCACGTTGTTCCTCTTTTGAGGAACAAGTTTTCTTGTCTGGAAGTTT	48
MS2-BARCODE1111-57	GCAGCTGGATTCCTCTTTTGAGGAACAAGTTTTCTTGTACGACAGACG	48
MS2-BARCODE1111-58	GCCATCTAACTCCTCTTTTGAGGAACAAGTTTTCTTGTTTGATGTTAG	48
MS2-BARCODE1111-59	TACCGACCTGTCCTCTTTTGAGGAACAAGTTTTCTTGTACGTACGGCT	48
MS2-BARCODE1111-60	CTCATAGGAATCCTCTTTTGAGGAACAAGTTTTCTTGTGAAACTCTTG	48
MS2-BARCODE1111-61	AAGGTGAACCTCCTCTTTTGAGGAACAAGTTTTCTTGTTTCGTAAGCA	48
MS2-BARCODE1111-62	TCTCATATGCACCCTGGATATCACTCATTAGT	32
MS2-BARCODE1111-63	GGTAACCAACCGAACTGCAACTCCAACCACCTGCCGGC	38
MS2-BARCODE1111-64	CACGTGTTTTGATCGAAACTTTCGATCTTCGTTTAGGG	38
MS2-BARCODE1111-65	CAAGGTAGCGGAGCGCCTGGCGCCAATTACCGCGACGA	38
MS2-BARCODE1111-66	GCGGCAGTGTACGCCTTCACGAGCGCAATGGTTTGCGT	38
MS2-BARCODE1111-67	CGCGAGTTGTGAGGCTGTCGACCTGGCCTCTGCTAAAG	38
MS2-BARCODE1111-68	CAACACCAAGGTTAAAATTACCCTGGGTGACCTTTTGC	38
MS2-BARCODE1111-69	AGGACTTCGGTCGACGCCCGGTTCGCAACGTTCTGCGG	38
MS2-BARCODE1111-70	CACTTCGATGTAAGTCAAGTTTTGGCTTACAGGGAAGA	38
MS2-BARCODE1111-71	GGCTGTAGCAGGAGCGTGCGTCGAGGGAGAAGCCGAAA	38
MS2-BARCODE1111-72	AGGCAGCCCGATCTATTTTATTATTCTTCGGAACTGTAAA	40
MS2-BARCODE1111-73	ACCCCTTTCTGGAGGTACATATTCATATCAGGCTCCTTAC	40
MS2-BARCODE1111-74	CATTCAGGTCTATACCAACGGATTTGAGCCGGCGTCTGATGAAAGCACCG	50
MS2-BARCODE1111-75	CGAACCATCTACGCTGCCCTGCTGAGCCAGACGCTGGTTGATCGATTGAT	50
MS2-BARCODE1111-76	AGGCGATCGGAGATGGAATCGGATGCAGACGATAAGTCTATCGTCGCAAG	50
MS2-BARCODE1111-77	GGATACGATCGAGATATGAATATAGCTCTGGTGGGAGAAAACTCCACACC	50
MS2-BARCODE1111-78	AAATAGTTCCCATCGTATCGTCTCGCCATCTACGATTCCGTAGTGTGAGC	50
MS2-BARCODE1111-79	CAGAATATCATGGACTCTAGCTCAAATGTGAACCCATTTCCCATTGTGGA	50
MS2-BARCODE1111-80	CTATGGTTCCGGCGTTACCAAAATGGATTTGGGTCGCTTTGACTATTGCC	50
MS2-BARCODE1111-81	CACACGGGGTGCAATCTCACTGGGACATATAATATCGTCCCCGTAGATGC	50
MS2-BARCODE1111-82	AACGTTTTACGAAGATTCGGTTTAAAACCGTAGTAGGCAAGTGCCTCTAG	50
MS2-BARCODE1111-83	CACCACGGTAAAAGTGCGCGCCGCAGCTCTCGCGAAAGAGCCCGGACACG	50
MS2-BARCODE1111-84	GGCGAAGAGATTGTCAACAGGTTTCTTGATGTAAAACGGTTTGACATCGA	50
MS2-BARCODE1111-85	ATACCTCCGACAACTCCCCAACCCCGTAGCCGATTTAATATCAGCATCAG	50

MS2-BARCODE1111-86	GCACCTGGGAGGAGAGCCGTACCCACACCTTATAGAGGCGTGGATCTGAC	50
MS2-BARCODE1111-87	GACTACGTAGTAGTCGGCAGCGAGGTCCGTCCCACCGAAGAACATCGAAG	50
MS2-BARCODE1111-88	AGCCGCCCGTACGGAGTCTTGGTGTATACCGAGACTGCCGTAGGCGGGCT	50
MS2-BARCODE1111-89	CTCGAGCGATACGAGCAAGACGGAAACCCGAGGTACGGGTATCCGCGAGC	50
MS2-BARCODE1111-90	CCACGCTATGTAGCGACCACTGTCGTGCTTTTCGCTGAAGAACTTGCGTT	50
MS2-BARCODE1111-91	CGCACGCCGGCGGACTTCATGCTGTCGGTGATTTCACCTCCAGTATGGAA	50
MS2-BARCODE1111-92	CCTGAGGGAATGTGGGAACCGGCGTTAGCCACTCCGAAGTGCGTATAACG	50
MS2-BARCODE1111-93	TTACGGGGGTCCCTCGGTCAGCTACCGAGGAGAGCTCGCTGGCCCACACT	50
MS2-BARCODE1111-94	GGAGCCGGACCGCTTTCGCACCCGTGCTCTTTCGAGCACACCCACCCCGT	50
MS2-BARCODE1111-95	TCCTCTCTTTAGGGGGAGGTCCCTGGGCCGAAGCCCGCCCACCTTTCGGT	50
MS2-BARCODE1111-96	TGGGTGGTAACTAGCCAAGCAGCTAGTTACCAAATCGGGAGAATCCCGGG	50

Table S3. Oligonucleotide sequences for DNA strands complementary to M13 DNA and producing the 001 DNA barcode. Dumbbell-containing sequences are highlighted in red.

NAME	SEQUENCE (5′ TO 3′)	LENGTH
1	TTTTCGTAATCATGGTCATAGCTGTTTCCTGTGTGAAATTGTTATC	46
2	CGCTCACAATTCCACACAACATACGAGCCGGAAGCATA	38
3	AAGTGTAAAGCCTGGGGTGCCTAATGAGTGAGCTAACT	38
4	CACATTAATTGCGTTGCGCTCACTGCCCGCTTTCCAGT	38
5	CGGGAAACCTGTCGTGCCAGCTGCATTAATGAATCGGC	38
6	CAACGCGCGGGGAGAGGCGGTTTGCGTATTGGGCGCCA	38
7	GGGTGGTTTTTCTTTTCACCAGTGAGACGGGCAACAGC	38
8	TGATTGCCCTTCACCGCCTGGCCCTGAGAGAGTTGCAG	38
9	CAAGCGGTCCACGCTGGTTTGCCCCAGCAGGCGAAAAT	38
10	CCTGTTTGATGGTGGTTCCGAAATCGGCAAAATCCCTT	38
11	ATAAATCAAAAGAATAGCCCGAGATAGGGTTGAGTGTT	38
12	GTTCCAGTTTGGAACAAGAGTCCACTATTAAAGAACGT	38
13	GGACTCCAACGTCAAAGGGCGAAAAACCGTCTATCAGG	38
14	GCGATGGCCCACTACGTGAACCATCACCCAAATCAAGT	38
15	TTTTTGGGGTCGAGGTGCCGTAAAGCACTAAATCGGAA	38
16	CCCTAAAGGGAGCCCCCGATTTAGAGCTTGACGGGGAA	38
17	AGCCGGCGAACGTGGCGAGAAAGGAAGGGAAGAAAGCG	38
18	AAAGGAGCGGGCGCTAGGGCGCTGGCAAGTGTAGCGGT	38
19	CACGCTGCGCGTAACCACCACACCCGCCGCGCTTAATG	38
20	CGCCGCTACAGGGCGCGTACTATGGTTGCTTTGACGAG	38
21	CACGTATAACGTGCTTTCCTCGTTAGAATCAGAGCGGG	38
22	AGCTAAACAGGAGGCCGATTAAAGGGATTTTAGACAGG	38
23	AACGGTACGCCAGAATCCTGAGAAGTGTTTTTATAATC	38
24	AGTGAGGCCACCGAGTAAAAGAGTCTGTCCATCACGCA	38
25	AATTAACCGTTGTAGCAATACTTCTTTGATTAGTAATA	38
26	ACATCACTTGCCTGAGTAGAAGAACTCAAACTATCGGC	38
27	CTTGCTGGTAATATCCAGAACAATATTACCGCCAGCCA	38
28	TTGCAACAGGAAAAACGCTCATGGAAATACCTACATTT	38

29	TGACGCTCAATCGTCTGAAATGGATTATTTACATTGGC	38
30	AGATTCACCAGTCACACGACCAGTAATAAAAGGGACAT	38
31	TCTGGCCAACAGAGATAGAACCCTTCTGACCTGAAAGC	38
32	GTAAGAATACGTGGCACAGACAATATTTTTGAATGGCT	38
33	ATTAGTCTTTAATGCGCGAACTGATAGCCCTAAAACAT	38
34	CGCCATTAAAAATACCGAACGAACCACCAGCAGAAGAT	38
35	AAAACAGAGGTGAGGCGGTCAGTATTAACACCGCCTGC	38
36	AACAGTGCCACGCTGAGAGCCAGCAGCAAATGAAAAAT	38
37	CTAAAGCATCACCTTGCTGAACCTCAAATATCAAACCC	38
38	TCAATCAATATCTGGTCAGTTGGCAAATCAACAGTTGA	38
39	AAGGAATTGAGGAAGGTTATCTAAAATATCTTTAGGAG	38
40	CACTAACAACTAATAGATTAGAGCCGTCAATAGATAAT	38
41	ACATTTGAGGATTTAGAAGTATTAGACTTTACAAACAA	38
42	TTCGACAACTCGTATTAAATCCTTTGCCCGAACGTTAT	38
43	TAATTTTAAAAGTTTGAGTAACATTATCATTTTGCGGA	38
44	ACAAAGAAACCACCAGAAGGAGCGGAATTATCATCATA	38
45	TTCCTGATTATCAGATGATGGCAATTCATCAATATAAT	38
46	CCTGATTGTTTGGATTATACTTCTGAATAATGGAAGGG	38
47	TTAGAACCTACCATATCAAAATTATTTGCACGTAAAAC	38
48	AGAAATAAAGAAATTGCGTAGATTTTCAGGTTTAACGT	38
49	CAGATGAATATACAGTAACAGTACCTTTTACATCGGGA	38
50	GAAACAATAACGGATTCGCCTGATTGCTTTGAATACCA	38
51	AGTTACAAAATCGCGCAGAGGCGAATTATTCATTTCAA	38
52	TTACCTGAGCAAAAGAAGATGATGAAACAAACATCAAG	38
53	AAAACAAAATTAATTACATTTAACAATTTCATTTGAAT	38
54	AATATAATCCTGATTGTTTGGATTATACTTCTGAATAATGGAAGGG	46
55	CACTAACAACTCCTCTTTTGAGGAACAAGTTTTCTTGTTAATAGATTA	48
56	GAGCCGTCAATCCTCTTTTGAGGAACAAGTTTTCTTGTTAGATAATAC	48
57	ATTTGAGGATTCCTCTTTTGAGGAACAAGTTTTCTTGTTTAGAAGTAT	48
58	TAGACTTTACTCCTCTTTTGAGGAACAAGTTTTCTTGTAAACAATTCG	48
59	ACAACTCGTATCCTCTTTTGAGGAACAAGTTTTCTTGTTTAAATCCTT	48
60	TGCCCGAACGTCCTCTTTTGAGGAACAAGTTTTCTTGTTTATTAATTT	48
61	GATTAAGACGTCCTCTTTTGAGGAACAAGTTTTCTTGTCTGAGAAGAG	48
62	TCAATAGTGATCCTCTTTTGAGGAACAAGTTTTCTTGTATTTATCAAA	48
63	ATCATAGGTCTCCTCTTTTGAGGAACAAGTTTTCTTGTTGAGAGACTA	48
64	CCTTTTTAACTCCTCTTTTGAGGAACAAGTTTTCTTGTCTCCGGCTTA	48
65	GGTTGGGTTATCCTCTTTTGAGGAACAAGTTTTCTTGTTATAACTATA	48
66	AACTTTTTCAAATATATTTTAGTTAATTTCATCTTCTG	38
67	ACCTAAATTTAATGGTTTGAAATACCGACCGTGTGATA	38
68	AATAAGGCGTTAAATAAGAATAAACACCGGAATCATAA	38
69	TTACTAGAAAAAGCCTGTTTAGTATCATATGCGTTATA	38
70	CAAATTCTTACCAGTATAAAGCCAACGCTCAACAGTAG	38
71	GGCTTAATTGAGAATCGCCATATTTAACAACGCCAACA	38
72	TGTAATTTAGGCAGAGGCATTTTCGAGCCAGTAATAAG	38
73	TCATCGAGAACAAGCAAGCCGTTTTTATTTTCATCGTAGGAATCAT	46

74	AGAATATAAAGTACCGACAAAAGGTAAAGTAATTCTGT	38
75	CCAGACGACGACAATAAACAACATGTTCAGCTAATGCA	38
76	GAACGCGCCTGTTTATCAACAATAGATAAGTCCTGAAC	38
77	AAGAAAAATAATATCCCATCCTAATTTACGAGCATGTA	38
78	GAAACCAATCAATAATCGGCTGTCTTTCCTTATCATTC	38
79	CAAGAACGGGTATTAAACCAAGTACCGCACTCATCGAG	38
80	AACAAGCAAGCCGTTTTTATTTTCATCGTAGGAATCAT	38
81	TTATCCGGTATTCTAAGAACGCGAGGCGTTTTAGCGAA	38
82	CCTCCCGACTTGCGGGAGGTTTTGAAGCCTTAAATCAA	38
83	GATTAGTTGCTATTTTGCACCCAGCTACAATTTTATCC	38
84	TGAATCTTACCAACGCTAACGAGCGTCTTTCCAGAGCC	38
85	TAATTTGCCAGTTACAAAATAAACAGCCATATTATTTA	38
86	TCCCAATCCAAATAAGAAACGATTTTTTGTTTAACGTC	38
87	AAAAATGAAAATAGCAGCCTTTACAGAGAGAATAACAT	38
88	AAAAACAGGGAAGCGCATTAGACGGGAGAATTAACTGA	38
89	ACACCCTGAACAAAGTCAGAGGGTAATTGAGCGCTAAT	38
90	ATCAGAGAGATAACCCACAAGAATTGAGTTAAGCCCAA	38
91	TAATAAGAGCAAGAAACAATGAAATAGCAATAGCTATC	38
92	TTACCGAAGCCCTTTTTAAGAAAAGTAAGCAGATAGCC	38
93	GAACAAAGTTACCAGAAGGAAACCGAGGAAACGCAATA	38
94	ATAACGGAATACCCAAAAGAACTGGCATGATTAAGACT	38
95	CCTTATTACGCAGTATGTTAGCAAACGTAGAAAATACA	38
96	TACATAAAGGTGGCAACATATAAAAGAAACGCAAAGAC	38
97	ACCACGGAATAAGTTTATTTTGTCACAATCAATAGAAA	38
98	ATTCATATGGTTTACCAGCGCCAAAGACAAAAGGGCGA	38
99	CATTCAACCGATTGAGGGAGGGAAGGTAAATATTGACG	38
100	GAAATTATTCATTAAAGGTGAATTATCACCGTCACCGA	38
101	CTTGAGCCATTTGGGAATTAGAGCCAGCAAAATCACCA	38
102	GTAGCACCATTACCATTAGCAAGGCCGGAAACGTCACC	38
103	AATGAAACCATCGATAGCAGCACCGTAATCAGTAGCGA	38
104	CAGAATCAAGTTTGCCTTTAGCGTCAGACTGTAGCGCG	38
105	TTTTCATCGGCATTTTCGGTCATAGCCCCCTTATTAGC	38
106	GTTTGCCATCTTTTCATAATCAAAATCACCGGAACCAG	38
107	AGCCACCACCGGAACCGCCTCCCTCAGAGCCGCCACCC	38
108	TCAGAACCGCCACCCTCAGAGCCACCACCCTCAGAGCC	38
109	GCCACCAGAACCACCACCAGAGCCGCCGCCAGCATTGA	38
110	CAGGAGGTTGAGGCAGGTCAGACGATTGGCCTTGATAT	38
111	TCACAAACAAATAAATCCTCATTAAAGCCAGAATGGAA	38
112	AGCGCAGTCTCTGAATTTACCGTTCCAGTAAGCGTCAT	38
113	ACATGGCTTTTGATGATACAGGAGTGTACTGGTAATAA	38
114	GTTTTAACGGGGTCAGTGCCTTGAGTAACAGTGCCCGT	38
115	ATAAACAGTTAATGCCCCCTGCCTATTTCGGAACCTAT	38
116	TATTCTGAAACATGAAAGTATTAAGAGGCTGAGACTCC	38
117	TCAAGAGAAGGATTAGGATTAGCGGGGTTTTGCTCAGT	38
118	ACCAGGCGGATAAGTGCCGTCGAGAGGGTTGATATAAG	38

119	TATAGCCCGGAATAGGTGTATCACCGTACTCAGGAGGT	38
120	TTAGTACCGCCACCCTCAGAACCGCCACCCTCAGAACC	38
121	GCCACCCTCAGAGCCACCACCCTCATTTTCAGGGATAG	38
122	CAAGCCCAATAGGAACCCATGTACCGTAACACTGAGTT	38
123	TCGTCACCAGTACAAACTACAACGCCTGTAGCATTCCA	38
124	CAGACAGCCCTCATAGTTAGCGTAACGATCTAAAGTTT	38
125	TGTCGTCTTTCCAGACGTTAGTAAATGAATTTTCTGTA	38
126	TGGGATTTTGCTAAACAACTTTCAACAGTTTCAGCGGA	38
127	GTGAGAATAGAAAGGAACAACTAAAGGAATTGCGAATA	38
128	ATAATTTTTTCACGTTGAAAATCTCCAAAAAAAAGGCT	38
129	CCAAAAGGAGCCTTTAATTGTATCGGTTTATCAGCTTG	38
130	CTTTCGAGGTGAATTTCTTAAACAGCTTGATACCGATA	38
131	GTTGCGCCGACAATGACAACAACCATCGCCCACGCATA	38
132	ACCGATATATTCGGTCGCTGAGGCTTGCAGGGAGTTAA	38
133	AGGCCGCTTTTGCGGGATCGTCACCCTCAGCAGCGAAA	38
134	GACAGCATCGGAACGAGGGTAGCAACGGCTACAGAGGC	38
135	TTTGAGGACTAAAGACTTTTTCATGAGGAAGTTTCCAT	38
136	TAAACGGGTAAAATACGTAATGCCACTACGAAGGCACC	38
137	AACCTAAAACGAAAGAGGCAAAAGAATACACTAAAACA	38
138	CTCATCTTTGACCCCCAGCGATTATACCAAGCGCGAAA	38
139	CAAAGTACAACGGAGATTTGTATCATCGCCTGATAAAT	38
140	TGTGTCGAAATCCGCGACCTGCTCCATGTTACTTAGCC	38
141	GGAACGAGGCGCAGACGGTCAATCATAAGGGAACCGAA	38
142	CTGACCAACTTTGAAAGAGGACAGATGAACGGTGTACA	38
143	GACCAGGCGCATAGGCTGGCTGACCTTCATCAAGAGTA	38
144	ATCTTGACAAGAACCGGATATTCATTACCCAAATCAAC	38
145	GTAACAAAGCTGCTCATTCAGTGAATAAGGCTTGCCCT	38
146	GACGAGAAACACCAGAACGAGTAGTAAATTGGGCTTGA	38
147	GATGGTTTAATTTCAACTTTAATCATTGTGAATTACCT	38
148	TATGCGATTTTAAGAACTGGCTCATTATACCAGTCAGG	38
149	ACGTTGGGAAGAAAAATCTACGTTAATAAAACGAACTA	38
150	ACGGAACAACATTATTACAGGTAGAAAGATTCATCAGT	38
151	TGAGATTTAGGAATACCACATTCAACTAATGCAGATAC	38
152	ATAACGCCAAAAGGAATTACGAGGCATAGTAAGAGCAA	38
153	CACTATCATAACCCTCGTTTACCAGACGACGATAAAAA	38
154	CCAAAATAGCGAGAGGCTTTTGCAAAAGAAGTTTTGCC	38
155	AGAGGGGGTAATAGTAAAATGTTTAGACTGGATAGCGT	38
156	CCAATACTGCGGAATCGTCATAAATATTCATTGAATCC	38
157	CCCTCAAATGCTTTAAACAGTTCAGAAAACGAGAATGA	38
158	CCATAAATCAAAAATCAGGTCTTTACCCTGACTATTAT	38
159	AGTCAGAAGCAAAGCGGATTGCATCAAAAAGATTAAGA	38
160	GGAAGCCCGATCCTCTTTTGAGGAACAAGTTTTCTTGTAAGACTTCAA	48
161	ATATCGCGTTTCCTCTTTTGAGGAACAAGTTTTCTTGTTTAATTCGAG	48
162	CTTCAAAGCGTCCTCTTTTGAGGAACAAGTTTTCTTGTAACCAGACCG	48
163	GAAGCAAACTTCCTCTTTTGAGGAACAAGTTTTCTTGTCCAACAGGTC	48

164	AGGATTAGAGTCCTCTTTTGAGGAACAAGTTTTCTTGTAGTACCTTTA	48
165	ATTGCTCCTTTCCTCTTTTGAGGAACAAGTTTTCTTGTTTGATAAGAG	48
166	GTCATTTTTGTCCTCTTTTGAGGAACAAGTTTTCTTGTCGGATGGCTT	48
167	AGAGCTTAATTCCTCTTTTGAGGAACAAGTTTTCTTGTTGCTGAATAT	48
168	AATGCTGTAGTCCTCTTTTGAGGAACAAGTTTTCTTGTCTCAACATGT	48
169	TTTAAATATGTCCTCTTTTGAGGAACAAGTTTTCTTGTCAACTAAAGT	48
170	ACGGTGTCTGTCCTCTTTTGAGGAACAAGTTTTCTTGTGAAGTTTCAT	48
171	TCCATATAACTCCTCTTTTGAGGAACAAGTTTTCTTGTAGTTGATTCC	48
172	CAATTCTGCGAACGAGTAGATTTAGT	26
173	GTTTAGCTATATTTTCATTTGGGGCGCGAGCTGAAAAG	38
174	GTGGCATCAATTCTACTAATAGTAGTAGCATTAACATC	38
175	CAATAAATCATACAGGCAAGGCAAAGAATTAGCAAAAT	38
176	TAAGCAATAAAGCCTCAGAGCATAAAGCTAAATCGGTT	38
177	GTACCAAAAACATTATGACCCTGTAATACTTTTGCGGG	38
178	AGAAGCCTTTATTTCAACGCAAGGATAAAAATTTTTAG	38
179	AACCCTCATATATTTTAAATGCAATGCCTGAGTAATGT	38
180	GTAGGTAAAGATTCAAAAGGGTGAGAAAGGCCGGAGAC	38
181	AGTCAAATCACCATCAATATGATATTCAACCGTTCTAG	38
182	CTGATAAATTAATGCCGGAGAGGGTAGCTATTTTTGAG	38
183	AGATCTACAAAGGCTATCAGGTCATTGCCTGAGAGTCT	38
184	GGAGCAAACAAGAGAATCGATGAACGGTAATCGTAAAA	38
185	CTAGCATGTCAATCATATGTACCCCGGTTGATAATCAG	38
186	AAAAGCCCCAAAAACAGGAAGATTGTATAAGCAAATAT	38
187	TTAAATTGTAAACGTTAATATTTTGTTAAAATTCGCAT	38
188	TAAATTTTTGTTAAATCAGCTCATTTTTTAACCAATAG	38
189	GAACGCCATCAAAAATAATTCGCGTCTGGCCTTCCTGT	38
190	AGCCAGCTTTCATCAACATTAAATGTGAGCGAGTAACA	38
191	ACCCGTCGGATTCTCCGTGGGAACAAACGGCGGATTGA	38
192	CCGTAATGGGATAGGTCACGTTGGTGTAGATGGGCGCA	38
193	TCGTAACCGTGCATCTGCCAGTTTGAGGGGACGACGAC	38
194	AGTATCGGCCTCAGGAAGATCGCACTCCAGCCAGCTTT	38
195	CCGGCACCGCTTCTGGTGCCGGAAACCAGGCAAAGCGC	38
196	CATTCGCCATTCAGGCTGCGCAACTGTTGGGAAGGGCG	38
197	ATCGGTGCGGGCCTCTTCGCTATTACGCCAGCTGGCGA	38
198	AAGGGGGATGTGCTGCAAGGCGATTAAGTTGGGTAACG	38
199	CCAGGGTTTTCCCAGTCACGACGTTGTAAAACGACGGC	38
200	CAGTGCCAAGCTTGCATGCCTGCAGGTCGACTCTAGAGGATCTTTT	46

Table S4. Oligonucleotide sequences for DNA strands complementary to M13 DNA and producing the 011 DNA barcode. Dumbbell-containing sequences are highlighted in red.

NAME	SEQUENCE (5′ TO 3′)	LENGTH
1	TTTTCGTAATCATGGTCATAGCTGTTTCCTGTGTGAAATTGTTATC	46

2	CGCTCACAATTCCACACAACATACGAGCCGGAAGCATA	38
3	AAGTGTAAAGCCTGGGGTGCCTAATGAGTGAGCTAACT	38
4	CACATTAATTGCGTTGCGCTCACTGCCCGCTTTCCAGT	38
5	CGGGAAACCTGTCGTGCCAGCTGCATTAATGAATCGGC	38
6	CAACGCGCGGGGAGAGGCGGTTTGCGTATTGGGCGCCA	38
7	GGGTGGTTTTTCTTTTCACCAGTGAGACGGGCAACAGC	38
8	TGATTGCCCTTCACCGCCTGGCCCTGAGAGAGTTGCAG	38
9	CAAGCGGTCCACGCTGGTTTGCCCCAGCAGGCGAAAAT	38
10	CCTGTTTGATGGTGGTTCCGAAATCGGCAAAATCCCTT	38
11	ATAAATCAAAAGAATAGCCCGAGATAGGGTTGAGTGTT	38
12	GTTCCAGTTTGGAACAAGAGTCCACTATTAAAGAACGT	38
13	GGACTCCAACGTCAAAGGGCGAAAAACCGTCTATCAGG	38
14	GCGATGGCCCACTACGTGAACCATCACCCAAATCAAGT	38
15	TTTTTGGGGTCGAGGTGCCGTAAAGCACTAAATCGGAA	38
16	CCCTAAAGGGAGCCCCCGATTTAGAGCTTGACGGGGAA	38
17	AGCCGGCGAACGTGGCGAGAAAGGAAGGGAAGAAAGCG	38
18	AAAGGAGCGGGCGCTAGGGCGCTGGCAAGTGTAGCGGT	38
19	CACGCTGCGCGTAACCACCACACCCGCCGCGCTTAATG	38
20	CGCCGCTACAGGGCGCGTACTATGGTTGCTTTGACGAG	38
21	CACGTATAACGTGCTTTCCTCGTTAGAATCAGAGCGGG	38
22	AGCTAAACAGGAGGCCGATTAAAGGGATTTTAGACAGG	38
23	AACGGTACGCCAGAATCCTGAGAAGTGTTTTTATAATC	38
24	AGTGAGGCCACCGAGTAAAAGAGTCTGTCCATCACGCA	38
25	AATTAACCGTTGTAGCAATACTTCTTTGATTAGTAATA	38
26	ACATCACTTGCCTGAGTAGAAGAACTCAAACTATCGGC	38
27	CTTGCTGGTAATATCCAGAACAATATTACCGCCAGCCA	38
28	TTGCAACAGGAAAAACGCTCATGGAAATACCTACATTT	38
29	TGACGCTCAATCGTCTGAAATGGATTATTTACATTGGC	38
30	AGATTCACCAGTCACACGACCAGTAATAAAAGGGACAT	38
31	TCTGGCCAACAGAGATAGAACCCTTCTGACCTGAAAGC	38
32	GTAAGAATACGTGGCACAGACAATATTTTTGAATGGCT	38
33	ATTAGTCTTTAATGCGCGAACTGATAGCCCTAAAACAT	38
34	CGCCATTAAAAATACCGAACGAACCACCAGCAGAAGAT	38
35	AAAACAGAGGTGAGGCGGTCAGTATTAACACCGCCTGC	38
36	AACAGTGCCACGCTGAGAGCCAGCAGCAAATGAAAAAT	38
37	CTAAAGCATCACCTTGCTGAACCTCAAATATCAAACCC	38
38	TCAATCAATATCTGGTCAGTTGGCAAATCAACAGTTGA	38
39	AAGGAATTGAGGAAGGTTATCTAAAATATCTTTAGGAG	38
40	CACTAACAACTAATAGATTAGAGCCGTCAATAGATAAT	38
41	ACATTTGAGGATTTAGAAGTATTAGACTTTACAAACAA	38
42	TTCGACAACTCGTATTAAATCCTTTGCCCGAACGTTAT	38
43	TAATTTTAAAAGTTTGAGTAACATTATCATTTTGCGGA	38
44	ACAAAGAAACCACCAGAAGGAGCGGAATTATCATCATA	38
45	TTCCTGATTATCAGATGATGGCAATTCATCAATATAAT	38
46	CCTGATTGTTTGGATTATACTTCTGAATAATGGAAGGG	38

47	TTAGAACCTACCATATCAAAATTATTTGCACGTAAAAC	38
48	AGAAATAAAGAAATTGCGTAGATTTTCAGGTTTAACGT	38
49	CAGATGAATATACAGTAACAGTACCTTTTACATCGGGA	38
50	GAAACAATAACGGATTCGCCTGATTGCTTTGAATACCA	38
51	AGTTACAAAATCGCGCAGAGGCGAATTATTCATTTCAA	38
52	TTACCTGAGCAAAAGAAGATGATGAAACAAACATCAAG	38
53	AAAACAAAATTAATTACATTTAACAATTTCATTTGAAT	38
54	AATATAATCCTGATTGTTTGGATTATACTTCTGAATAATGGAAGGG	46
55	CACTAACAACTCCTCTTTTGAGGAACAAGTTTTCTTGTTAATAGATTA	48
56	GAGCCGTCAATCCTCTTTTGAGGAACAAGTTTTCTTGTTAGATAATAC	48
57	ATTTGAGGATTCCTCTTTTGAGGAACAAGTTTTCTTGTTTAGAAGTAT	48
58	TAGACTTTACTCCTCTTTTGAGGAACAAGTTTTCTTGTAAACAATTCG	48
59	ACAACTCGTATCCTCTTTTGAGGAACAAGTTTTCTTGTTTAAATCCTT	48
60	TGCCCGAACGTCCTCTTTTGAGGAACAAGTTTTCTTGTTTATTAATTT	48
61	GATTAAGACGTCCTCTTTTGAGGAACAAGTTTTCTTGTCTGAGAAGAG	48
62	TCAATAGTGATCCTCTTTTGAGGAACAAGTTTTCTTGTATTTATCAAA	48
63	ATCATAGGTCTCCTCTTTTGAGGAACAAGTTTTCTTGTTGAGAGACTA	48
64	CCTTTTTAACTCCTCTTTTGAGGAACAAGTTTTCTTGTCTCCGGCTTA	48
65	GGTTGGGTTATCCTCTTTTGAGGAACAAGTTTTCTTGTTATAACTATA	48
66	AACTTTTTCAAATATATTTTAGTTAATTTCATCTTCTG	38
67	ACCTAAATTTAATGGTTTGAAATACCGACCGTGTGATA	38
68	AATAAGGCGTTAAATAAGAATAAACACCGGAATCATAA	38
69	TTACTAGAAAAAGCCTGTTTAGTATCATATGCGTTATA	38
70	CAAATTCTTACCAGTATAAAGCCAACGCTCAACAGTAG	38
71	GGCTTAATTGAGAATCGCCATATTTAACAACGCCAACA	38
72	TGTAATTTAGGCAGAGGCATTTTCGAGCCAGTAATAAG	38
73	TCATCGAGAACAAGCAAGCCGTTTTTATTTTCATCGTAGGAATCAT	46
74	AGAATATAAATCCTCTTTTGAGGAACAAGTTTTCTTGTGTACCGACAA	48
75	AAGGTAAAGTTCCTCTTTTGAGGAACAAGTTTTCTTGTAATTCTGTCC	48
76	AGACGACGACTCCTCTTTTGAGGAACAAGTTTTCTTGTAATAAACAAC	48
77	ATGTTCAGCTTCCTCTTTTGAGGAACAAGTTTTCTTGTAATGCAGAAC	48
78	GCGCCTGTTTTCCTCTTTTGAGGAACAAGTTTTCTTGTATCAACAATA	48
79	GATAAGTCCTTCCTCTTTTGAGGAACAAGTTTTCTTGTGAACAAGAAA	48
80	AATAATATCCTCCTCTTTTGAGGAACAAGTTTTCTTGTCATCCTAATT	48
81	TACGAGCATGTCCTCTTTTGAGGAACAAGTTTTCTTGTTAGAAACCAA	48
82	TCAATAATCGTCCTCTTTTGAGGAACAAGTTTTCTTGTGCTGTCTTTC	48
83	CTTATCATTCTCCTCTTTTGAGGAACAAGTTTTCTTGTCAAGAACGGG	48
84	TATTAAACCATCCTCTTTTGAGGAACAAGTTTTCTTGTAGTACCGCAC	48
85	TTATCCGGTATTCTAAGAACGCGAGGCGTTTTAGCGAA	38
86	CCTCCCGACTTGCGGGAGGTTTTGAAGCCTTAAATCAA	38
87	GATTAGTTGCTATTTTGCACCCAGCTACAATTTTATCC	38
88	TGAATCTTACCAACGCTAACGAGCGTCTTTCCAGAGCC	38
89	TAATTTGCCAGTTACAAAATAAACAGCCATATTATTTA	38
90	TCCCAATCCAAATAAGAAACGATTTTTTGTTTAACGTC	38
91	AAAAATGAAAATAGCAGCCTTTACAGAGAGAATAACAT	38

92	AAAAACAGGGAAGCGCATTAGACGGGAGAATTAACTGA	38
93	ACACCCTGAACAAAGTCAGAGGGTAATTGAGCGCTAAT	38
94	ATCAGAGAGATAACCCACAAGAATTGAGTTAAGCCCAA	38
95	TAATAAGAGCAAGAAACAATGAAATAGCAATAGCTATC	38
96	TTACCGAAGCCCTTTTTAAGAAAAGTAAGCAGATAGCC	38
97	GAACAAAGTTACCAGAAGGAAACCGAGGAAACGCAATA	38
98	ATAACGGAATACCCAAAAGAACTGGCATGATTAAGACT	38
99	CCTTATTACGCAGTATGTTAGCAAACGTAGAAAATACA	38
100	TACATAAAGGTGGCAACATATAAAAGAAACGCAAAGAC	38
101	ACCACGGAATAAGTTTATTTTGTCACAATCAATAGAAA	38
102	ATTCATATGGTTTACCAGCGCCAAAGACAAAAGGGCGA	38
103	CATTCAACCGATTGAGGGAGGGAAGGTAAATATTGACG	38
104	GAAATTATTCATTAAAGGTGAATTATCACCGTCACCGA	38
105	CTTGAGCCATTTGGGAATTAGAGCCAGCAAAATCACCA	38
106	GTAGCACCATTACCATTAGCAAGGCCGGAAACGTCACC	38
107	AATGAAACCATCGATAGCAGCACCGTAATCAGTAGCGA	38
108	CAGAATCAAGTTTGCCTTTAGCGTCAGACTGTAGCGCG	38
109	TTTTCATCGGCATTTTCGGTCATAGCCCCCTTATTAGC	38
110	GTTTGCCATCTTTTCATAATCAAAATCACCGGAACCAG	38
111	AGCCACCACCGGAACCGCCTCCCTCAGAGCCGCCACCC	38
112	TCAGAACCGCCACCCTCAGAGCCACCACCCTCAGAGCC	38
113	GCCACCAGAACCACCACCAGAGCCGCCGCCAGCATTGA	38
114	CAGGAGGTTGAGGCAGGTCAGACGATTGGCCTTGATAT	38
115	TCACAAACAAATAAATCCTCATTAAAGCCAGAATGGAA	38
116	AGCGCAGTCTCTGAATTTACCGTTCCAGTAAGCGTCAT	38
117	ACATGGCTTTTGATGATACAGGAGTGTACTGGTAATAA	38
118	GTTTTAACGGGGTCAGTGCCTTGAGTAACAGTGCCCGT	38
119	ATAAACAGTTAATGCCCCCTGCCTATTTCGGAACCTAT	38
120	TATTCTGAAACATGAAAGTATTAAGAGGCTGAGACTCC	38
121	TCAAGAGAAGGATTAGGATTAGCGGGGTTTTGCTCAGT	38
122	ACCAGGCGGATAAGTGCCGTCGAGAGGGTTGATATAAG	38
123	TATAGCCCGGAATAGGTGTATCACCGTACTCAGGAGGT	38
124	TTAGTACCGCCACCCTCAGAACCGCCACCCTCAGAACC	38
125	GCCACCCTCAGAGCCACCACCCTCATTTTCAGGGATAG	38
126	CAAGCCCAATAGGAACCCATGTACCGTAACACTGAGTT	38
127	TCGTCACCAGTACAAACTACAACGCCTGTAGCATTCCA	38
128	CAGACAGCCCTCATAGTTAGCGTAACGATCTAAAGTTT	38
129	TGTCGTCTTTCCAGACGTTAGTAAATGAATTTTCTGTA	38
130	TGGGATTTTGCTAAACAACTTTCAACAGTTTCAGCGGA	38
131	GTGAGAATAGAAAGGAACAACTAAAGGAATTGCGAATA	38
132	ATAATTTTTTCACGTTGAAAATCTCCAAAAAAAAGGCT	38
133	CCAAAAGGAGCCTTTAATTGTATCGGTTTATCAGCTTG	38
134	CTTTCGAGGTGAATTTCTTAAACAGCTTGATACCGATA	38
135	GTTGCGCCGACAATGACAACAACCATCGCCCACGCATA	38
136	ACCGATATATTCGGTCGCTGAGGCTTGCAGGGAGTTAA	38

137	AGGCCGCTTTTGCGGGATCGTCACCCTCAGCAGCGAAA	38
138	GACAGCATCGGAACGAGGGTAGCAACGGCTACAGAGGC	38
139	TTTGAGGACTAAAGACTTTTTCATGAGGAAGTTTCCAT	38
140	TAAACGGGTAAAATACGTAATGCCACTACGAAGGCACC	38
141	AACCTAAAACGAAAGAGGCAAAAGAATACACTAAAACA	38
142	CTCATCTTTGACCCCCAGCGATTATACCAAGCGCGAAA	38
143	CAAAGTACAACGGAGATTTGTATCATCGCCTGATAAAT	38
144	TGTGTCGAAATCCGCGACCTGCTCCATGTTACTTAGCC	38
145	GGAACGAGGCGCAGACGGTCAATCATAAGGGAACCGAA	38
146	CTGACCAACTTTGAAAGAGGACAGATGAACGGTGTACA	38
147	GACCAGGCGCATAGGCTGGCTGACCTTCATCAAGAGTA	38
148	ATCTTGACAAGAACCGGATATTCATTACCCAAATCAAC	38
149	GTAACAAAGCTGCTCATTCAGTGAATAAGGCTTGCCCT	38
150	GACGAGAAACACCAGAACGAGTAGTAAATTGGGCTTGA	38
151	GATGGTTTAATTTCAACTTTAATCATTGTGAATTACCT	38
152	TATGCGATTTTAAGAACTGGCTCATTATACCAGTCAGG	38
153	ACGTTGGGAAGAAAAATCTACGTTAATAAAACGAACTA	38
154	ACGGAACAACATTATTACAGGTAGAAAGATTCATCAGT	38
155	TGAGATTTAGGAATACCACATTCAACTAATGCAGATAC	38
156	ATAACGCCAAAAGGAATTACGAGGCATAGTAAGAGCAA	38
157	CACTATCATAACCCTCGTTTACCAGACGACGATAAAAA	38
158	CCAAAATAGCGAGAGGCTTTTGCAAAAGAAGTTTTGCC	38
159	AGAGGGGGTAATAGTAAAATGTTTAGACTGGATAGCGT	38
160	CCAATACTGCGGAATCGTCATAAATATTCATTGAATCC	38
161	CCCTCAAATGCTTTAAACAGTTCAGAAAACGAGAATGA	38
162	CCATAAATCAAAAATCAGGTCTTTACCCTGACTATTAT	38
163	AGTCAGAAGCAAAGCGGATTGCATCAAAAAGATTAAGA	38
164	GGAAGCCCGATCCTCTTTTGAGGAACAAGTTTTCTTGTAAGACTTCAA	48
165	ATATCGCGTTTCCTCTTTTGAGGAACAAGTTTTCTTGTTTAATTCGAG	48
166	CTTCAAAGCGTCCTCTTTTGAGGAACAAGTTTTCTTGTAACCAGACCG	48
167	GAAGCAAACTTCCTCTTTTGAGGAACAAGTTTTCTTGTCCAACAGGTC	48
168	AGGATTAGAGTCCTCTTTTGAGGAACAAGTTTTCTTGTAGTACCTTTA	48
169	ATTGCTCCTTTCCTCTTTTGAGGAACAAGTTTTCTTGTTTGATAAGAG	48
170	GTCATTTTTGTCCTCTTTTGAGGAACAAGTTTTCTTGTCGGATGGCTT	48
171	AGAGCTTAATTCCTCTTTTGAGGAACAAGTTTTCTTGTTGCTGAATAT	48
172	AATGCTGTAGTCCTCTTTTGAGGAACAAGTTTTCTTGTCTCAACATGT	48
173	TTTAAATATGTCCTCTTTTGAGGAACAAGTTTTCTTGTCAACTAAAGT	48
174	ACGGTGTCTGTCCTCTTTTGAGGAACAAGTTTTCTTGTGAAGTTTCAT	48
175	TCCATATAACTCCTCTTTTGAGGAACAAGTTTTCTTGTAGTTGATTCC	48
176	CAATTCTGCGAACGAGTAGATTTAGT	26
177	GTTTAGCTATATTTTCATTTGGGGCGCGAGCTGAAAAG	38
178	GTGGCATCAATTCTACTAATAGTAGTAGCATTAACATC	38
179	CAATAAATCATACAGGCAAGGCAAAGAATTAGCAAAAT	38
180	TAAGCAATAAAGCCTCAGAGCATAAAGCTAAATCGGTT	38
181	GTACCAAAAACATTATGACCCTGTAATACTTTTGCGGG	38

182	AGAAGCCTTTATTTCAACGCAAGGATAAAAATTTTTAG	38
183	AACCCTCATATATTTTAAATGCAATGCCTGAGTAATGT	38
184	GTAGGTAAAGATTCAAAAGGGTGAGAAAGGCCGGAGAC	38
185	AGTCAAATCACCATCAATATGATATTCAACCGTTCTAG	38
186	CTGATAAATTAATGCCGGAGAGGGTAGCTATTTTTGAG	38
187	AGATCTACAAAGGCTATCAGGTCATTGCCTGAGAGTCT	38
188	GGAGCAAACAAGAGAATCGATGAACGGTAATCGTAAAA	38
189	CTAGCATGTCAATCATATGTACCCCGGTTGATAATCAG	38
190	AAAAGCCCCAAAAACAGGAAGATTGTATAAGCAAATAT	38
191	TTAAATTGTAAACGTTAATATTTTGTTAAAATTCGCAT	38
192	TAAATTTTTGTTAAATCAGCTCATTTTTTAACCAATAG	38
193	GAACGCCATCAAAAATAATTCGCGTCTGGCCTTCCTGT	38
194	AGCCAGCTTTCATCAACATTAAATGTGAGCGAGTAACA	38
195	ACCCGTCGGATTCTCCGTGGGAACAAACGGCGGATTGA	38
196	CCGTAATGGGATAGGTCACGTTGGTGTAGATGGGCGCA	38
197	TCGTAACCGTGCATCTGCCAGTTTGAGGGGACGACGAC	38
198	AGTATCGGCCTCAGGAAGATCGCACTCCAGCCAGCTTT	38
199	CCGGCACCGCTTCTGGTGCCGGAAACCAGGCAAAGCGC	46
200	CATTCGCCATTCAGGCTGCGCAACTGTTGGGAAGGGCG	38
201	ATCGGTGCGGGCCTCTTCGCTATTACGCCAGCTGGCGA	38
202	AAGGGGGATGTGCTGCAAGGCGATTAAGTTGGGTAACG	38
203	CCAGGGTTTTCCCAGTCACGACGTTGTAAAACGACGGC	38
204	CAGTGCCAAGCTTGCATGCCTGCAGGTCGACTCTAGAGGATCTTTT	46

11. References

(1) Bell, N. A. W.; Keyser, U. F. Digitally encoded DNA nanostructures for multiplexed, single-molecule protein sensing with nanopores. *Nature Nanotechnology* **2016**, *11* (7), 645-651. DOI: 10.1038/nnano.2016.50.

(2) Patiño-Guillén, G.; Pešović, J.; Panić, M.; Savić-Pavićević, D.; Bošković, F.; Keyser, U. F. Single-molecule RNA sizing enables quantitative analysis of alternative transcription termination. *Nature Communications* **2024**, *15* (1), 1699. DOI: 10.1038/s41467-024-45968-8.

(3) Hawkins, T. L.; Mirigian, M.; Li, J.; Yasar, M. S.; Sackett, D. L.; Sept, D.; Ross, J. L. Perturbations in microtubule mechanics from tubulin preparation. *Cellular and Molecular Bioengineering* **2012**, *5*, 227-238.

(4) Beuwer, M. A.; Knopper, M. F.; Albertazzi, L.; van der Zwaag, D.; Ellenbroek, W. G.; Meijer, E. W.; Prins, M. W. J.; Zijlstra, P. Mechanical properties of single supramolecular polymers from correlative AFM and fluorescence microscopy. *Polymer Chemistry* **2016**, *7* (47), 7260-7268, 10.1039/C6PY01656A. DOI: 10.1039/C6PY01656A.

(5) Kumar Sharma, R.; Agrawal, I.; Dai, L.; Doyle, P. S.; Garaj, S. Complex DNA knots detected with a nanopore sensor. *Nature Communications* **2019**, *10* (1), 4473. DOI: 10.1038/s41467-019-12358-4.
